# Incremental value of risk factor variability for cardiovascular risk prediction in individuals with type 2 diabetes: results from UK primary care electronic health records

**DOI:** 10.1093/ije/dyac140

**Published:** 2022-07-01

**Authors:** Zhe Xu, Matthew Arnold, Luanluan Sun, David Stevens, Ryan Chung, Samantha Ip, Jessica Barrett, Stephen Kaptoge, Lisa Pennells, Emanuele Di Angelantonio, Angela M Wood

**Affiliations:** 1 British Heart Foundation Cardiovascular Epidemiology Unit, Department of Public Health and Primary Care, University of Cambridge, Cambridge, UK; 2 Medical Research Council Biostatistics Unit, Cambridge Institute of Public Health, University of Cambridge, Cambridge, UK; 3 National Institute for Health Research Blood and Transplant Research Unit in Donor Health and Genomics, University of Cambridge, Cambridge, UK; 4 British Heart Foundation Centre of Research Excellence, University of Cambridge, Cambridge, UK; 5 Health Data Research UK Cambridge, Wellcome Genome Campus and University of Cambridge, Cambridge, UK; 6 The Alan Turing Institute, London, UK

**Keywords:** Cardiovascular disease, risk prediction, type 2 diabetes, variability, repeated measurements, electronic health records

## Abstract

**Background:**

Cardiovascular disease (CVD) risk prediction models for individuals with type 2 diabetes are important tools to guide intensification of interventions for CVD prevention. We aimed to assess the added value of incorporating risk factors variability in CVD risk prediction for people with type 2 diabetes.

**Methods:**

We used electronic health records (EHRs) data from 83 910 adults with type 2 diabetes but without pre-existing CVD from the UK Clinical Practice Research Datalink for 2004–2017. Using a landmark-modelling approach, we developed and validated sex-specific Cox models, incorporating conventional predictors and trajectories plus variability of systolic blood pressure (SBP), total and high-density lipoprotein (HDL) cholesterol, and glycated haemoglobin (HbA_1c_). Such models were compared against simpler models using single last observed values or means.

**Results:**

The standard deviations (SDs) of SBP, HDL cholesterol and HbA_1c_ were associated with higher CVD risk (P<0.05). Models incorporating trajectories and variability of continuous predictors demonstrated improvement in risk discrimination (C-index = 0.659, 95% CI: 0.654–0.663) as compared with using last observed values (C-index = 0.651, 95% CI: 0.646–0.656) or means (C-index = 0.650, 95% CI: 0.645–0.655). Inclusion of SDs of SBP yielded the greatest improvement in discrimination (C-index increase = 0.005, 95% CI: 0.004-0.007) in comparison to incorporating SDs of total cholesterol (C-index-increase = 0.002, 95% CI: 0.000–0.003), HbA_1c_ (C-index increase = 0.002, 95% CI: 0.000–0.003) or HDL cholesterol (C-index increase= 0.003,95% CI: 0.002–0.005).

**Conclusion:**

Incorporating variability of predictors from EHRs provides a modest improvement in CVD risk discrimination for individuals with type 2 diabetes. Given that repeat measures are readily available in EHRs especially for regularly monitored patients with diabetes, this improvement could easily be achieved.

## Introduction

The prevalence of co-morbidities amongst people with type 2 diabetes is increasing in the UK^1^ and globally,^2^ and presents challenges to effective diabetes management.^1^ Cardiovascular disease (CVD) is a major cause of death or disability among people with type 2 diabetes^3^ and adults with type 2 diabetes have about a 2-fold excess risk of developing CVD, independently from other established CVD risk factors.^4^ Identifying those at highest risk of CVD at early stages is fundamental for CVD prevention.^3^ Furthermore, CVD risk assessment is important for guiding intensification of interventions and setting treatment goals for blood pressure, lipid, and glucose in people with type 2 diabetes.^5^ To this end, CVD risk prediction models specifically for individuals with type 2 diabetes have been developed^6–9^ and recommended for clinical use in several national guidelines.^10–12^ However, most models use single measurements of risk predictors^8^ and only a few [e.g. the United Kingdom Prospective Diabetes Study (UKPDS)^13,14^] incorporate repeat measures through the use of means. Additionally, increased variability in risk factors [e.g. systolic blood pressure (SBP), cholesterol, glycated haemoglobin (HbA_1c_)] is associated with increased CVD risk, independently of the factors themselves,^15–17^ and may be considered as additional components in risk assessment models. Investigation of the potential gains of using variability of risk factors in longitudinal data for CVD risk prediction among type 2 diabetes people is needed.

The benefits of using electronic health records (EHRs) for CVD risk assessment and subsequent personalised healthcare decisions are well recognised.^18^ Such benefits may be greater amongst the diabetes population due to higher frequencies of routine health assessments than the general population. Therefore, the aim of this study was to evaluate whether the utilisation of within-person variability of repeatedly measured predictors from a large population-representative EHRs database could provide additional value to CVD risk prediction among individuals with type 2 diabetes in comparison with standard models.

## Methods

### Data source and study population

The Clinical Practice Research Datalink (CPRD) is a longitudinal primary care database of anonymised EHRs from the UK general practitioners. It covers approximately 6.9% of the UK population and is representative of the UK primary care setting with respect to age, sex, and ethnicity structure.^19^ We also used linked data from Hospital Episode Statistics (HES) and Office for National Statistics (ONS). In this research, we used data restricted to the England region due to the linkage availability.

For this proposed analysis, individuals entered the study on the latest of following dates: the date of 6 months after registration at the general practice; the date the individual turned 40 years old (note, prior measurements of CVD predictors from age ≥30 years were extracted for these individuals); the date that the data for the practice were up to standard; ^20^ or 1 April 2004, the date of introduction of the Quality and Outcomes Framework.^21^ Individuals were followed up until the earliest date of the following: the individual’s death or first CVD event; the date that the individual turned 85 years old (note, follow-up data up to age 95 years were extracted for these individuals); the date of deregistration at the practice; the last contact date for the practice with CPRD; or 30 November 2017, end of data availability.

Among the 2 589 074 individuals with linked data during study entry and exit dates, we identified 159 730 individuals with confirmed diagnosed type 2 diabetes (diagnosis codes^22^ listed in Supplementary Appendix 1, available as Supplementary data at *IJE* online) at study entry or during follow-up but without prevalent CVD events at study entry. We further excluded people who experienced incident CVD events during follow-up prior to the diagnosis of diabetes. Since our primary aim was to compare models using single vs repeated predictors and their variability, we restricted our study population to those with complete information on risk predictor variables (listed below). Thus, the analysis data set consisted of 83 910 individuals (flowchart in Supplementary Figure S1, available as Supplementary data at *IJE* online). We randomly split our data by practice to a 2/3 derivation data set (263 practices with 53 292 individuals) and a 1/3 validation data set (132 practices with 30 618 individuals).

### Outcome

The outcome of interest was incident CVD, where CVD was defined as fatal or non-fatal coronary heart disease (including myocardial infarction and angina), stroke or transient ischaemic attack. The definition matched the QRISK algorithm,^23^ which is recommended by the current UK CVD risk assessment guidelines.^24^ We ascertained incident CVD to be the first CVD event in any of the three databases (CPRD, HES or ONS Death registry). Code lists are provided in Supplementary Appendix 2 (available as Supplementary data at *IJE* online).

### Predictor variables

Based on the most commonly used predictor variables in existing CVD risk prediction tools among people with diabetes^6–8,13,14^ (e.g. UKPDS^13,14^) and the availability of our routine EHRs data, we selected the following predictors: diabetes duration, SBP, total cholesterol, high-density lipoprotein (HDL) cholesterol, HbA_1c_ (for which details of measurements have been previously described^19^), smoking status (current smoker or not ascertained from CPRD Read codes), blood pressure-lowering medication (yes/no ascertained from CPRD prescription information), previous diagnoses of atrial fibrillation (yes/no ascertained from CPRD Read codes and HES ICD-10 codes) and ethnicity (White, Asian, Black, mixed, other or unspecified/missing, ascertained from CPRD Read codes). We did not include statin use as a predictor since it is not commonly used in existing models and a large proportion of individuals with diabetes already initiated statins (Supplementary Figure S2, available as Supplementary data at *IJE* online).

### Statistical analysis

**Figure 1:**
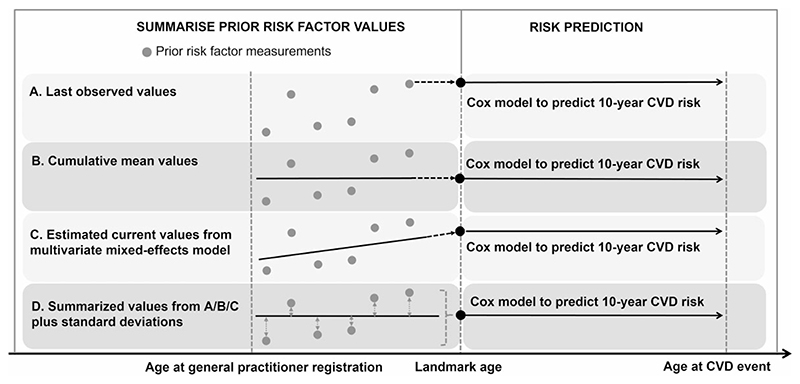
Schematic of using repeated measurements of risk factors to predict 10-year cardiovascular disease risk among people with type 2 diabetes. Individuals contribute their electronic health records data at one or more landmark ages if they are without pre-existing CVD and have complete information on risk factors measured before that landmark age.

We used a landmark approach^25,26^ to leverage the longitudinal risk predictor values recorded in EHRs (Figure 1). We defined landmark ages at 40, 41, 42, …, 85 years as entry points at which we predicted 10-year CVD risk based on risk predictor values recorded prior to that age.^26^ We used a two-stage approach to derive the prediction model in a 2/3 derivation data set. In Stage 1, at each landmark age, we summarised all prior available assessments from age 30 onwards for SBP, total and HDL cholesterols, and HbA_1c_ by either the last observed values, the cumulative crude means or their estimated ‘current’ values (i.e. the values at the landmark age) and ‘individual-level slope’ (i.e. the degree of change in the risk predictor over time) calculated from age-specific and sex-specific multivariate mixed-effects linear regression models with fixed and random age-varying effects (details in Supplementary Appendix 3, available as Supplementary data at *IJE* online). The standard deviations (SDs) of these risk factors were further calculated to quantify the within-person variability among individuals with at least two prior measurements. Duration of diabetes was defined as the time since age at diabetes diagnosis (calculated based on the earliest date of the ascertainment of diabetes) to each landmark age; smoking status was defined from the last observed values at each landmark age; blood pressure-lowering medication use and atrial fibrillation were defined as ever having a related prescription or diagnosis record before the landmark age.

In Stage 2, sex-specific ‘super landmark’ Cox models^25,26^ using the stacked data across all landmark ages with robust standard errors were used to derive the 10-year CVD risk prediction model. In addition to the predictors estimated from Stage 1, we included landmark age, landmark age-squared and landmark age interaction terms with SBP, total cholesterol, HDL cholesterol, HbA1c, and smoking status in the Cox model. The proportional hazards assumption was tested by examining the Schoenfeld residuals and we did not observe a clear indication of a violation of the assumption in our models. Estimated hazard ratios and the baseline hazard functions from the Cox model were then applied to the 1/3 validation data set to estimate 10-year CVD risk.

In the validation data set, we compared the predictive performance of the Cox models using last observed values, mean values or estimated current values from multivariate mixed-effects models, with and without individual-level slopes and SDs of risk factors. The predictive performance for models with SDs of risk was assessed in subgroups of individuals with at least 2, 3, 4, …, ≥10 measurements of each predictor. Discrimination was compared using Harrell’s C-index; ^27^ calibration was assessed quantitatively by calculating the calibration slope;^27,28^ predictive accuracy was evaluated using the Brier scores;^29^ reclassification was measured using the continuous net reclassification improvement (NRI).^30^ Supplementary Appendix 4 (available as Supplementary data at *IJE* online) provides details of these performance evaluation metrics.

In sensitivity analyses, we refitted the multivariate mixed-effects linear regression models and super landmark models amongst 143 466 individuals who had at least one measurement of any risk factors of SBP, total cholesterol, HDL cholesterol, HbA_1c_ or smoking status (Supplementary Figure S1, available as Supplementary data at *IJE* online).

All statistical analyses were conducted using Stata version 15.1 (StataCorp LLC, College Station, Texas) and R version 3.6.1 (R Foundation for Statistical Computing, Vienna, Austria). Statistical significance was defined using a two-sided P-value of <0.05. This study follows the TRIPOD reporting guideline (Supplementary Appendix 5, available as Supplementary data at *IJE* online).

## Results

### Characteristics of study population

Among the 83 910 people in the analysis data set, 32 804 (39%) were women and the mean age at type 2 diabetes recorded diagnosis was 59.3 (SD = 12.0) years. Characteristics at study entry in the derivation and validation data set were similar (Table 1). In total, there were 12 298 incident CVD events during a median follow-up period of 8.98 (interquartile range: 5.32, 11.55) years (Supplementary Figures S3 and S4, available as Supplementary data at *IJE* online). Individuals with complete data included in our analysis were slightly older, more likely to be male and had lower CVD incidence rate than those with missing risk predictor measurements (17.5 vs 21.6 per 1000 person-years, respectively) (Supplementary Table S1, available as Supplementary data at *IJE* online). A large proportion (83%) of people had at least two prior measures of each continuous risk predictor, especially measures of SBP, total cholesterol, and HbA1c, and 57% had at least five measures (Supplementary Figures S5 and S6, available as Supplementary data at *IJE* online).

### Risk predictor levels and associations with incident CVD in people with type 2 diabetes

Estimated current values of SBP from mixed-effects models were slightly higher than last observed values but lower than the means; estimated current values of total cholesterol were similar to the last observed values but lower than the means (Supplementary Figure S7, available as Supplementary data at *IJE* online).

Hazard ratios (HRs) for each of SBP, HDL total cholesterol, and HbA_1c_ were broadly similar in models using either last observed, mean or estimated current values (Supplementary Tables S2 and S3, available as Supplementary data at *IJE* online). Importantly, the SDs of SBP, HDL cholesterol, and HbA1c were strongly associated with higher CVD risk (Supplementary Tables S4 and S5, available as Supplementary data at *IJE* online). The individual-level slopes (i.e. representing the change in risk predictor values over time) of SBP and cholesterol were not associated with incident CVD and not considered further (Supplementary Table S6, available as Supplementary data at *IJE* online).

### Model performance comparison

**Figure 2:**
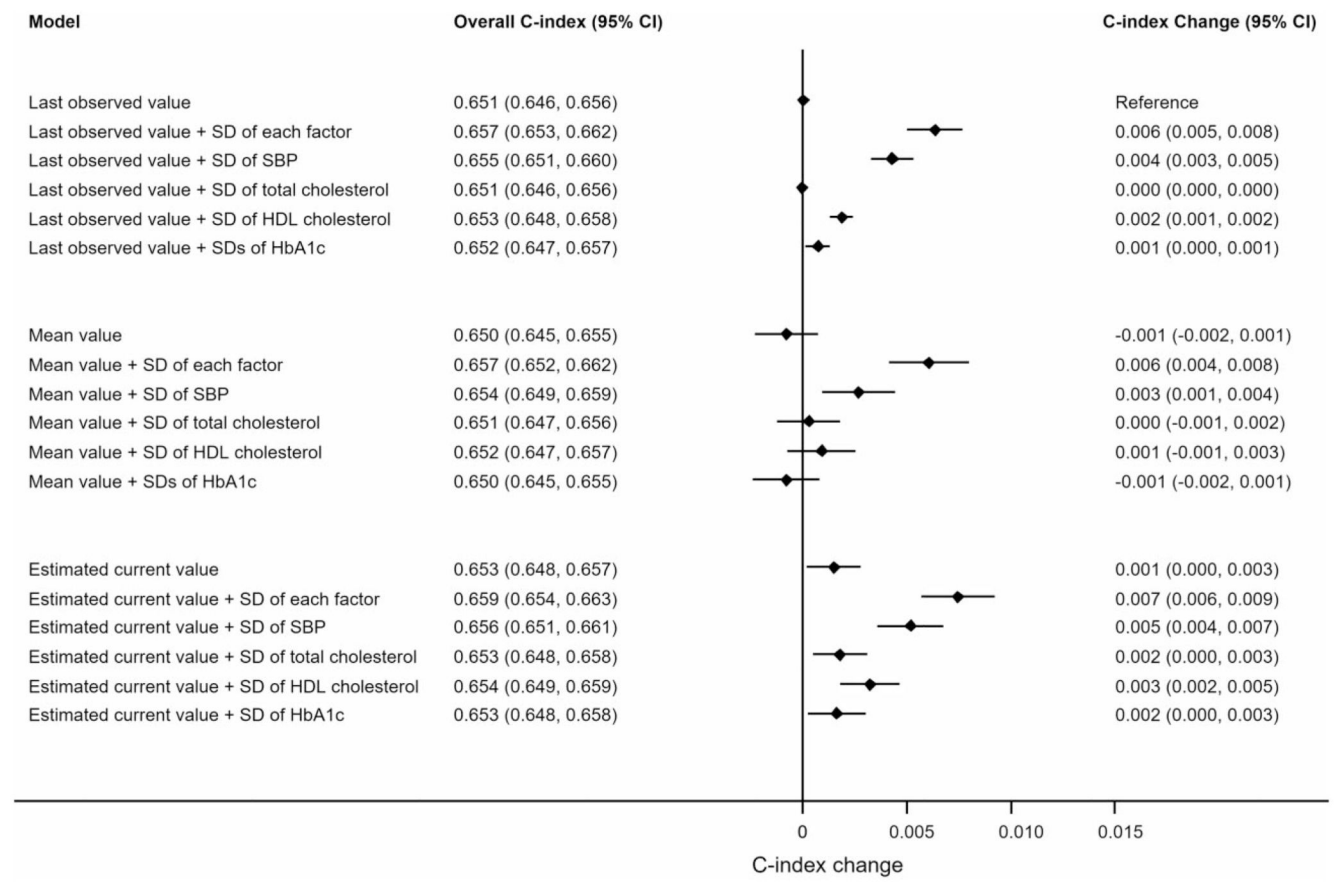
Change in cardiovascular disease risk discrimination between models in the validation data set for people with at least two repeat measures of systolic blood pressure (SBP) and total cholesterol and high-density lipoprotein (HDL) cholesterol and glycated haemoglobin (HbA_1c_), Clinical Practice Research Datalink, Hospital Episode Statistics and the Office for National Statistics, England, United Kingdom, 2004–2017. **Last observed value model**: Sex-specific Cox regression model with estimated risk factor values of SBP, total cholesterol, HDL cholesterol, and HbA_1c_ from **last observed values**, together with landmark age, landmark age-squared, ethnicity, duration of diabetes, smoking status, blood pressure-lowering medication use, and atrial fibrillation status, plus landmark age interaction terms with SBP, total cholesterol, HDL cholesterol, HbA_1c_, and smoking status. **Last observed value** + **SD model**: Last observed value model plus **standard deviations** of SBP, total cholesterol, HDL cholesterol, and HbA_1c_ as indicated. **Mean value model**: Sex-specific Cox regression model with estimated risk factor values of SBP, total cholesterol, HDL cholesterol, and HbA_1c_ from **cumulative means**,together with risk factors and interaction terms as noted in the last observed value model. **Mean value** + **SD model**: Mean value model plus **standard deviations** of SBP, total cholesterol, HDL cholesterol, and HbA_1c_ as indicated. **Estimated current value model**: Sex-specific Cox regression model with **estimated current risk factor values** of SBP, total cholesterol, HDL cholesterol, and HbA_1c_ from **multivariate mixed-effects linear regression models**, together with risk factors and interaction terms as noted in the last observed value model. **Estimated current value** + **SD model**: Estimated current value model plus **standard deviations** of SBP, total cholesterol, HDL cholesterol, and HbA_1c_ as indicated.

Using last observed values of predictors, the C-index of the prediction model was 0.652 (95% CI: 0.647, 0.656) (Table 2). Replacing the last observed values with the estimated current values slightly improved discrimination in the validation data set, with a C-index increase of 0.001 (95% CI: 0.000, 0.002) (Table 2). Model discrimination also increased with the addition of SDs of SBP, total and HDL cholesterol, and HbA_1c_ to all risk prediction models (Figure 2). Compared with the model using last observed values and without SDs, the model using estimated current values with SDs increased the C-index by 0.007 (95% CI: 0.006, 0.009) (Figure 2). This C-index increase was comparable with or even greater than the added benefit from well-established predictors (e.g. SBP, total cholesterol, HDL cholesterol) in our study population. For example, including SBP improved the C-index by 0.007 (95% CI: 0.005, 0.008) compared with the Cox model without SBP; including total and HDL cholesterol improved the C-index by 0.004 (95% CI: 0.003, 0.006) compared with the Cox model without cholesterol (Supplementary Table S7, available as Supplementary data at *IJE* online). Further investigations that added the SDs of each risk predictor separately revealed that the SDs of SBP yielded the strongest impact on discriminative improvement with a largest C-index increase of 0.005 (95% CI: 0.004, 0.007) compared with results from adding SDs of total cholesterol (C-index increase = 0.002, 95% CI: 0.000, 0.003), HDL cholesterol (C-index increase = 0.003, 95% CI: 0.002, 0.005) or HbA_1c_ (C-index increase = 0.002, 95% CI: 0.000, 0.003) (Figure 2), and the results were generally consistent amongst subgroups of people with different numbers of repeated measurements (Supplementary Figures S8–S12, available as Supplementary data at *IJE* online). Improvements in the C-index were slightly greater in men compared with women (Supplementary Figures S13 and S14, available as Supplementary data at *IJE* online).

Brier scores estimated using different approaches were similar (Table 2), with lower values observed on the addition of the risk factor SDs (Supplementary Table S8 and Supplementary Figure S15, available as Supplementary data at *IJE* online). In contrast to a fairly constant C-index with increasing numbers of risk factor measurements (Supplementary Figure S12, available as Supplementary data at *IJE* online), we observed curve-linear relationships between the Brier score and the number of risk factor measurements (Supplementary Figure S15, available as Supplementary data at *IJE* online). Calibration slopes by age ranged from 0.17 to 1.67 and were approximately 1 between ages 60 and 70 years, and those values deviating from 1 were mostly observed in women at younger or older ages with relatively fewer CVD events (Supplementary Figures S16 and S17, available as Supplementary data at *IJE* online). No substantial improvements in NRI were observed across landmark ages when using the estimated current values compared with the last observed values (Supplementary Tables S9 and S10, available as Supplementary data at *IJE* online).

Sensitivity analyses on 143 466 (90%) individuals with at least one measurement of any risk factors of SBP, total cholesterol, HDL cholesterol, HbA_1c_ or smoking status produced similar HRs (Supplementary Table S11, available as Supplementary data at *IJE* online) to those reported above for 83 910 individuals with complete data on all risk factors. The overall C-index among this population was 0.670 (95% CI: 0.667, 0.673) (Supplementary Table S12, available as Supplementary data at *IJE* online).

## Discussion

The current analysis of 83 910 people with type 2 diabetes reliably assessed the added benefit of incorporating variability of repeatedly measured SBP, cholesterol, and HbA_1c_ from longitudinal EHRs into CVD risk prediction models. We found no improvement in CVD risk prediction when merely replacing single last observed measurements of SBP, cholesterol, and HbA_1c_ with means or current values estimated from prior longitudinal measurements. However, we did find a moderate discriminative improvement when incorporating the within-person variability (quantified by SDs) of past SBP, cholesterol, and HbA_1c_. Such improvement from longitudinal information was comparable with or even greater than the gains from well-established predictors (e.g. SBP and cholesterol) in our study population.

Our results concord with previous studies identifying independent associations of long-term within-person variability of SBP, HDL cholesterol, and HbA_1c_ with CVD risk in patients with type 2 diabetes^15–17^ and provide further support for better understanding their roles in CVD risk prediction, especially for use in readily available EHRs.^31^ Notably, due to differences in measurement feasibility of various predictors in general practitioners, more repeated SBP data are generally recorded compared with other CVD predictors. The larger number of measurements improves the precision of the SD (i.e. a simple SD estimated on 10 measurements is more accurate than an estimation on 2 measurements) and reduces the bias from measurement error and regression dilution. This is one possible explanation for why we found a greater discriminative improvement when adding the SD from SBP compared with less frequently measured predictors. Indeed, there are emerging methods that improve on the simple ‘standard deviation’ approximations of individual-level long-term and short-term variability.^32,33^ Such methods account for differing numbers of observations between risk predictions and individuals, and use more complex models to reduce the bias from regression dilution, which warrant further investigation.

Evidence from systematic reviews^6–8^ indicated that previous research rarely considered the added value of repeated measurements or long-term variability of predictors in CVD risk prediction among individuals with type 2 diabetes. Most studies used single baseline values and only a few applied the mean values of the past measurements [e.g. the UKPDS models^13,14^ and the Basque Country Prospective Complications and Mortality Study risk engine (BASCORE)^34^]. Several methods for using repeated measurements in CVD risk prediction such as joint models,^35^ landmark models^26^ and univariate mixed-effects models^36^ have been developed for the general population, but not specifically for the population with diabetes. These studies suggested that using repeated measures was associated with only slight to modest improvements in risk prediction.^31,37^ We observed similar findings in the current analyses when replacing the last observed values with the estimates from longitudinal models to predict CVD risk for people with type 2 diabetes people, who have relatively higher CVD risk and with more frequently collected repeated measures than the general population. However, after adding the SDs, the improvement in discrimination became stronger.

To our knowledge, this is the first study to investigate and compare methodologies incorporating longitudinal measurements of multiple risk predictors together, especially including HbA_1c_ and their SDs into CVD risk prediction for people with type 2 diabetes. The added benefit in model discriminative improvement may help to better guide the implementation of intensive therapeutic interventions for type 2 diabetes patients with highest CVD risk. Such predictive ability gain may also be informative for setting serial treatment targets of blood pressure, lipid, and glucose control based on CVD risk levels to help diabetes management. The use of national representative EHRs data, the large sample size, the high number of CVD events and long period of follow-up time further enhanced the reliability of the study results for the 10-year CVD risk prediction. Moreover, the landmark framework optimised the use of repeated measurements of risk predictors by age and enabled the estimation of age-varying risk predictor levels.

This study also has several potential limitations. First, Cox models were derived amongst those with complete data on risk predictors to make direct comparisons on the use of repeated measurements with single last observed values or mean values of risk factors and assess the added value of within-person variability. This may restrict the generalisability of the results to people with missing values, who may also have different characteristics (e.g. younger individuals are more likely to have missing values). Results of our sensitivity analyses from fitting the multivariate-mixed-effects models to people with at least one observed value of any risk factor (accounting for 90% of all individuals and thus including those with much sparser risk factor information) showed reasonable discriminatory performance. However, further work is needed to fully assess the value of within-person variability in these individuals. Second, the multivariate-mixed-effects model required additional assumptions (e.g. multivariate normal distribution for risk predictors) than simply using the last observed values or means. However, it has the advantage of handling missing values that are common in EHRs and enables the extension of our methods to a larger population with incomplete information. Third, the overall C-index in our analyses ranged from 0.65 to 0.67, which does not present an outstanding discriminative ability. Previous metaanalysis of CVD risk prediction models developed for people with diabetes also demonstrated similar results with a pooled C-index of 0.67 (95% CI: 0.66, 0.69).^8^ The poor discrimination may be partly due to people with diabetes being more homogeneous with shared characteristics for higher CVD risk than the general population. Therefore, it might be difficult to achieve high discrimination. This underlines the need to identify and incorporate new biomarkers for diabetes patients and to investigate risk models for recurrent CVD events risk in this ‘high-risk’ population in future prediction work.

## Conclusions

Our study highlighted the benefit of utilizing information from longitudinal past measurements of SBP, cholesterol, and HbA_1c_ to improve CVD risk prediction and guiding the intensitification of therapeutic interventions for people with type 2 diabetes. Incorporating the within-person variability of risk predictors provides a moderate improvement in CVD risk discrimination for individuals with type 2 diabetes. With the increasing availability and improving quality for routinely collected EHRs data, our approach may be easy and efficient to apply based on data already existing in EHRs. The added information from longitudinal risk predictor measurements can be integrated into future risk prediction models and help to improve CVD prevention as well as diabetes management for the type 2 diabetes population.

## Supplementary Material

Supplementary data are available at *IJE* online.

Supplementary File

## Figures and Tables

**Table 1:** Characteristics of 83 910 participants with type 2 diabetes included in the current study^a^

Characteristics	Derivation (*n* = 53 292)		Validation (*n* = 30 618)	
	Men (*n* = 32 639)	Women (*n* = 20 653)	Men (*n* = 18 467)	Women (*n* = 12 151)
Age at diagnosis of type 2 diabetes (years) [mean (SD)]	58.7 (11.5)	60.1 (12.5)	58.8 (11.6)	60.3 (12.6)
SBP^b^ (mmHg) [mean (SD)]	141.9 (18.9)	141.3 (20.1)	141.9 (19.2)	141.3 (20.4)
Total cholesterol^b^ (mmol/L) [mean (SD)]	5.0 (1.3)	5.4 (1.3)	5.0 (1.3)	5.4 (1.3)
HDL cholesterol^†^ (mmol/L) [mean (SD)]	1.2 (0.3)	1.3 (0.4)	1.2 (0.3)	1.3 (0.4)
HbA_1c_^b^ (%) [mean (SD)]	7.9 (2.0)	7. 7 (2.0)	7.9 (2.0)	7.7 (2.0)
No. of repeated measures of SBP per person [median (IQR)]	18 (9–28)	20 (11–31)	18 (9–29)	20 (11–32)
No. of repeated measures of total cholesterol per person [median (IQR)]	8 (5–13)	9 (5–13)	9 (5–13)	9 (5–14)
No. of repeated measures of HDL cholesterol per person [median (IQR)]	7 (3–11)	7 (4–11)	7 (4–11)	7 (4–12)
No. of repeated measures of HbA_1c_ per person [median (IQR)]	9 (5–15)	10 (5–16)	9 (5–16)	10 (5–16)
Current/ever smoker^c^ [*n* (%)]	12 240 (37.5)	8113 (39.3)	6916 (37.5)	4753 (39.2)
Ethnicity [*n* (%)]				
White	11 353 (34.8)	7775 (37.7)	6140 (33.3)	4376 (36.0)
Asian	1143 (3.5)	324 (1.6)	709 (3.8)	204 (1.7)
Black	627 (1.9)	325 (1.6)	366 (2.0)	173 (1.5)
Mixed	128 (0.4)	71 (0.3)	70 (0.4)	45 (0.4)
Other	291 (0.9)	91 (0.4)	210 (1.1)	78 (0.7)
Unspecified/missing	19 097 (58.5)	12 067 (58.4)	10 981 (59.4)	7268 (59.8)
History of prescription for antihypertensive medication^d^ [*n* (%)]	16 081 (49.3)	12 335 (59.7)	8877 (48.1)	7145 (58.8)
History of atrial fibrillation^d^ [*n* (%)]	811 (2.5)	403 (2.0)	478 (2.6)	261 (2.2)

CVD, cardiovascular disease; HDL, high-density lipoprotein; HbA_1c_, glycated haemoglobin; SBP, systolic blood pressure; SD, standard deviation.

a Included 83 910 individuals from Clinical Practice Research Datalink, Hospital Episode Statistics, and the Office for National Statistics, England, UK, 2004–2017, aged 40–85 years, without prevalent CVD before study entry, with confirmed type 2 diabetes before incident CVD events (if any) and/or study exit, and complete data on measurements of SBP, total cholesterol, HDL cholesterol, HbA_1c_, and smoking status between their study entry and study exit dates.

b Calculated using the first measurement values taken after study entry.

c Recorded as ‘yes’ if any of the measurement values showed ‘yes’ during follow-up.

d Recorded as ‘yes’ if any of the measurement values showed ‘yes’ before study entry.

**Table 2:** C-index and Brier score (with changes) for cardiovascular disease risk prediction for people with type 2 diabetes in the validation data set, Clinical Practice Research Datalink, Hospital Episode Statistics and the Office for National Statistics, England, UK, 2004–2017

Model	Overall^a^		Men		Women	
	C-index (95% CI)	Difference (95% CI)	C-index (95% CI)	Difference (95% CI)	C-index (95% CI)	Difference (95% CI)
Last observed value^b^	0.652 (0.647, 0.656)	Referent	0.652 (0.646, 0.657)	Referent	0.651 (0.643, 0.658)	Referent
Mean value^c^	0.650 (0.646, 0.655)	–0.002 (–0.003, 0.000)	0.650 (0.644, 0.656)	–0.002 (–0.003, 0.000)	0.650 (0.642, 0.657)	–0.002 (–0.004,0.001)
Estimated current value^d^	0.652 (0.648, 0.657)	0.001 (0.000, 0.002)	0.653 (0.647, 0.658)	0.001 (0.000, 0.003)	0.652 (0.643, 0.658)	0.000 (–0.002, 0.002)
	Brier score	Difference	Brier score	Difference	Brier score	Difference
	(95% CI)	(95% CI)	(95% CI)	(95% CI)	(95% CI)	(95% CI)
Last observed value	0.341 (0.338, 0.344)	Referent	0.338 (0.334, 0.342)	Referent	0.346 (0.340, 0.351)	Referent
Mean value	0.341 (0.338, 0.343)	–0.000 (–0.000, –0.000)	0.337(0.334, 0.341)	–0.000 (–0.000, –0.000)	0.346 (0.341, 0.352)	0.001 (0.001, 0.001)
Estimated current value	0.341 (0.339, 0.344)	0.000 (0.000, 0.000)	0.338 (0.334, 0.342)	0.000 (0.000, 0.000)	0.347 (0.341, 0.352)	0.001 (0.001, 0.001)

Higher C-index = greater risk discrimination; lower Brier score = better accuracy. HDL, high-density lipoprotein; HbA_1c_, glycated haemoglobin; SBP, systolic blood pressure.

a Overall C-index was calculated using combined data of the predicted risk for men and women; predicted risk was estimated from sex-specific Cox models.

b Last observed value: sex-specific Cox regression model with estimated risk factor values of SBP, total cholesterol, HDL cholesterol, and HbA_1c_ from **last observed values**, together with landmark age, landmark age-squared, ethnicity, duration of diabetes, smoking status, blood pressure-lowering medication use and atrial fibrillation status, plus landmark age interaction terms with SBP, total cholesterol, HDL cholesterol, HbA_1c_, and smoking status.

c Mean value: sex-specific Cox regression model with estimated risk factor values of SBP, total cholesterol, HDL cholesterol, and HbA_1c_ from **cumulative means**, together with risk factors and interaction terms as noted in the last observed value model.

d Estimated current value: sex-specific Cox regression model with **estimated current risk factor values** of SBP, total cholesterol, HDL cholesterol, and HbA_1c_ from **multivariate mixed-effects linear regression models**, together with risk factors and interaction terms as noted in the last observed value model.

## Data Availability

All data files are available from the CPRD databases. Z.X., M.A., D.S. and A.M.W. had access to the data and can take responsibility for the integrity of the data and the accuracy of the data analysis.

## Key Messages

- Cardiovascular disease (CVD) risk prediction models for individuals with type 2 diabetes are important tools to guide intensification of interventions for CVD prevention and diabetes management. However, most models use single measurements of risk predictors.
- Greater variability in risk predictors is associated with higher CVD risk, independently of the factors predictors themselves, and may be considered as additional components in risk assessment models for individuals with diabetes.
- We assessed the added value of incorporating within-person variability of repeatedly measured predictors into CVD risk prediction models for people with type 2 diabetes. These individuals are regularly monitored in primary care and have more repeated measurements than the general population.
- We derived and validated CVD risk prediction models on 83 910 individuals with type 2 diabetes from a large population-representative electronic health records database.
- Incorporating variability of repeat predictors of systolic blood pressure, total and high-density lipoprotein cholesterol, and glycated haemoglobin by using standard deviations provides a modest improvement in CVD risk discrimination for individuals with type 2 diabetes.
