# Supplementary material for: Incremental value of risk factor variability for cardiovascular risk prediction in individuals with type 2 diabetes: results from UK primary care electronic health records
Source: Int J Epidemiol. Author manuscript; Available in PMC 2022 Dec 15. (PMC9749723; doi:10.1093/ije/dyac140)
Supplement: Supplementary File [file EMS157107-supplement-Supplementary_File.pdf]

Supplementary Material

Variability in risk factors improves cardiovascular risk prediction for individuals with type 2 diabetes: results from UK primary care electronic health records

Zhe Xu, Matthew Arnold, Luanluan Sun, David Stevens, Ryan Chung, Samantha Ip, Jessica Barrett, Stephen Kaptoge, Lisa Pennells, Emanuele Di Angelantonio, and Angela M. Wood

Contents

Supplementary Appendix 1: Code list of type 2 diabetes 1

Supplementary Appendix 2: Code list of cardiovascular disease 4

Supplementary Appendix 3: Summarising repeated measures of risk predictors using multivariate mixed-effects linear regression models 10

Supplementary Appendix 4: Summary of metrics used for model predictive performance assessment 13

Supplementary Appendix 5: TRIPOD checklist 14

Supplementary Appendix 6: Supplementary tables and figures 16

References 50

Supplementary Appendix 1: Code list of type 2 diabetes

Type 2 diabetes was identified using diagnoses information from both primary care data in Clinical Practice Research Datalink (CPRD) and hospital admission data in Hospital Episode Statistics (HES), together with age at diagnosis, and medication prescription information.[1,2] In CPRD, diagnoses are coded using the hierarchical Read code system;[3] in the linked HES data, the International Classification of Disease 10th revision (ICD-10) code (ICD-10 = E11) were used.[4] Diabetes medication prescriptions information was extracted from CPRD product data.[5] Code list for CPRD diabetes diagnoses data is provided as follows:

Read code for CPRD data	
Read code	Description
C100112	Non-insulin dependent diabetes mellitus
C103y00	Other specified diabetes mellitus with coma
C109.00	Non-insulin dependent diabetes mellitus
C109000	Non-insulin-dependent diabetes mellitus with renal comps
C109011	Type II diabetes mellitus with renal complications
C109012	Type 2 diabetes mellitus with renal complications
C109100	Non-insulin-dependent diabetes mellitus with ophthalm comps
C109.11	NIDDM - Non-insulin dependent diabetes mellitus
C109111	Type II diabetes mellitus with ophthalmic complications
C109112	Type 2 diabetes mellitus with ophthalmic complications
C109.12	Type 2 diabetes mellitus
C109.13	Type II diabetes mellitus
C109200	Non-insulin-dependent diabetes mellitus with neuro comps
C109211	Type II diabetes mellitus with neurological complications
C109212	Type 2 diabetes mellitus with neurological complications
C109300	Non-insulin-dependent diabetes mellitus with multiple comps
C109312	Type 2 diabetes mellitus with multiple complications
C109400	Non-insulin dependent diabetes mellitus with ulcer
C109411	Type II diabetes mellitus with ulcer
C109412	Type 2 diabetes mellitus with ulcer
C109500	Non-insulin dependent diabetes mellitus with gangrene
C109511	Type II diabetes mellitus with gangrene
C109512	Type 2 diabetes mellitus with gangrene
C109600	Non-insulin-dependent diabetes mellitus with retinopathy
C109611	Type II diabetes mellitus with retinopathy
C109612	Type 2 diabetes mellitus with retinopathy
C109700	Non-insulin dependent diabetes mellitus - poor control
C109711	Type II diabetes mellitus - poor control
C109712	Type 2 diabetes mellitus - poor control
C109900	Non-insulin-dependent diabetes mellitus without complication
C109911	Type II diabetes mellitus without complication
C109912	Type 2 diabetes mellitus without complication
C109A00	Non-insulin dependent diabetes mellitus with mononeuropathy

C109A11	Type II diabetes mellitus with mononeuropathy
C109B00	Non-insulin dependent diabetes mellitus with polyneuropathy
C109B11	Type II diabetes mellitus with polyneuropathy
C109B12	Type 2 diabetes mellitus with polyneuropathy
C109C00	Non-insulin dependent diabetes mellitus with nephropathy
C109C11	Type II diabetes mellitus with nephropathy
C109C12	Type 2 diabetes mellitus with nephropathy
C109D00	Non-insulin dependent diabetes mellitus with hypoglyca coma
C109D11	Type II diabetes mellitus with hypoglycaemic coma
C109D12	Type 2 diabetes mellitus with hypoglycaemic coma
C109E00	Non-insulin depend diabetes mellitus with diabetic cataract
C109E11	Type II diabetes mellitus with diabetic cataract
C109E12	Type 2 diabetes mellitus with diabetic cataract
C109F11	Type II diabetes mellitus with peripheral angiopathy
C109F12	Type 2 diabetes mellitus with peripheral angiopathy
C109G00	Non-insulin dependent diabetes mellitus with arthropathy
C109G11	Type II diabetes mellitus with arthropathy
C109G12	Type 2 diabetes mellitus with arthropathy
C109H11	Type II diabetes mellitus with neuropathic arthropathy
C109H12	Type 2 diabetes mellitus with neuropathic arthropathy
C109J00	Insulin treated Type 2 diabetes mellitus
C109J11	Insulin treated non-insulin dependent diabetes mellitus
C109J12	Insulin treated Type II diabetes mellitus
C109K00	Hyperosmolar non-ketotic state in type 2 diabetes mellitus
C10F.00	Type 2 diabetes mellitus
C10F000	Type 2 diabetes mellitus with renal complications
C10F011	Type II diabetes mellitus with renal complications
C10F100	Type 2 diabetes mellitus with ophthalmic complications
C10F.11	Type II diabetes mellitus
C10F111	Type II diabetes mellitus with ophthalmic complications
C10F200	Type 2 diabetes mellitus with neurological complications
C10F211	Type II diabetes mellitus with neurological complications
C10F300	Type 2 diabetes mellitus with multiple complications
C10F311	Type II diabetes mellitus with multiple complications
C10F400	Type 2 diabetes mellitus with ulcer
C10F411	Type II diabetes mellitus with ulcer
C10F500	Type 2 diabetes mellitus with gangrene
C10F511	Type II diabetes mellitus with gangrene
C10F600	Type 2 diabetes mellitus with retinopathy
C10F611	Type II diabetes mellitus with retinopathy
C10F700	Type 2 diabetes mellitus - poor control
C10F711	Type II diabetes mellitus - poor control
C10F900	Type 2 diabetes mellitus without complication

C10F911	Type II diabetes mellitus without complication
C10FA00	Type 2 diabetes mellitus with mononeuropathy
C10FA11	Type II diabetes mellitus with mononeuropathy
C10FB00	Type 2 diabetes mellitus with polyneuropathy
C10FB11	Type II diabetes mellitus with polyneuropathy
C10FC00	Type 2 diabetes mellitus with nephropathy
C10FC11	Type II diabetes mellitus with nephropathy
C10FD00	Type 2 diabetes mellitus with hypoglycaemic coma
C10FD11	Type II diabetes mellitus with hypoglycaemic coma
C10FE00	Type 2 diabetes mellitus with diabetic cataract
C10FE11	Type II diabetes mellitus with diabetic cataract
C10FF00	Type 2 diabetes mellitus with peripheral angiopathy
C10FF11	Type II diabetes mellitus with peripheral angiopathy
C10FG11	Type II diabetes mellitus with arthropathy
C10FH00	Type 2 diabetes mellitus with neuropathic arthropathy
C10FH11	Type II diabetes mellitus with neuropathic arthropathy
C10FJ00	Insulin treated Type 2 diabetes mellitus
C10FJ11	Insulin treated Type II diabetes mellitus
C10FK00	Hyperosmolar non-ketotic state in type 2 diabetes mellitus
C10FK11	Hyperosmolar non-ketotic state in type II diabetes mellitus
C10FL00	Type 2 diabetes mellitus with persistent proteinuria
C10FL11	Type II diabetes mellitus with persistent proteinuria
C10FM00	Type 2 diabetes mellitus with persistent microalbuminuria
C10FM11	Type II diabetes mellitus with persistent microalbuminuria
C10FN00	Type 2 diabetes mellitus with ketoacidosis
C10FN11	Type II diabetes mellitus with ketoacidosis
C10FP00	Type 2 diabetes mellitus with ketoacidotic coma
C10FQ00	Type 2 diabetes mellitus with exudative maculopathy
C10FR00	Type 2 diabetes mellitus with gastroparesis

Supplementary Appendix 2: Code list of cardiovascular disease

Cardiovascular disease was defined as a combination of newly diagnoses of nonfatal or fatal events of coronary heart disease (CHD) (including myocardial infarction and angina), stroke, and transient ischemic attack (TIA), in line with the definition used in the QRISK3 CVD risk score.[3] In Clinical Practice Research Datalink (CPRD), diagnoses are coded using the hierarchical Read code system[3] and in the linked HES and ONS datasets, the International Classification of Disease 10th revision (ICD-10) codes were used.[4]

Read code for CPRD data	
Read code	Description
G3...00	Ischaemic heart disease
G31..00	Arteriosclerotic heart disease
G32..00	Atherosclerotic heart disease
G33..00	IHD - Ischaemic heart disease
G30..00	Acute myocardial infarction
G301.00	Attack - heart
G302.00	Coronary thrombosis
G303.00	Cardiac rupture following myocardial infarction (MI)
G304.00	Heart attack
G305.00	MI - acute myocardial infarction
G306.00	Thrombosis - coronary
G307.00	Silent myocardial infarction
G309800	Coronary thrombosis
G309900	Myocardial Infarction
G300.00	Acute anterolateral infarction
G301.00	Other specified anterior myocardial infarction
G301000	Acute anteroapical infarction
G301100	Acute anteroseptal infarction
G301z00	Anterior myocardial infarction NOS
G302.00	Acute inferolateral infarction
G303.00	Acute inferoposterior infarction
G304.00	Posterior myocardial infarction NOS
G305.00	Lateral myocardial infarction NOS
G306.00	True posterior myocardial infarction
G307.00	Acute subendocardial infarction
G307000	Acute non-Q wave infarction
G307100	Acute non-ST segment elevation myocardial infarction
G308.00	Inferior myocardial infarction NOS
G309.00	Acute Q-wave infarct
G30A.00	Mural thrombosis
G30B.00	Acute posterolateral myocardial infarction
G30X.00	Acute transmural myocardial infarction of unspecif site
G30X000	Acute ST segment elevation myocardial infarction
G30y.00	Other acute myocardial infarction
G30y000	Acute atrial infarction
G30y100	Acute papillary muscle infarction

G30y200	Acute septal infarction
G30yz00	Other acute myocardial infarction NOS
G30z.00	Acute myocardial infarction NOS
G31..00	Other acute and subacute ischaemic heart disease
G319900	Acute/subacute IHD NOS
G310.00	Postmyocardial infarction syndrome
G310100	Dressler's syndrome
G311.00	Preinfarction syndrome
G311100	Crescendo angina
G311200	Impending infarction
G311300	Unstable angina
G311400	Angina at rest
G311000	Myocardial infarction aborted
G311010	MI - myocardial infarction aborted
G311100	Unstable angina
G311200	Angina at rest
G311300	Refractory angina
G311400	Worsening angina
G311500	Acute coronary syndrome
G311z00	Preinfarction syndrome NOS
G312.00	Coronary thrombosis not resulting in myocardial infarction
G31y.00	Other acute and subacute ischaemic heart disease
G31y000	Acute coronary insufficiency
G31y099	Acute coronary syndrome
G31y100	Microinfarction of heart
G31y200	Subendocardial ischaemia
G31y300	Transient myocardial ischaemia
G31yz00	Other acute and subacute ischaemic heart disease NOS
G32..00	Old myocardial infarction
G321.00	Healed myocardial infarction
G322.00	Personal history of myocardial infarction
G33..00	Angina pectoris
G330.00	Angina decubitus
G330000	Nocturnal angina
G330z00	Angina decubitus NOS
G331.00	Prinzmetal's angina
G331100	Variant angina pectoris
G332.00	Coronary artery spasm
G33z.00	Angina pectoris NOS
G33z000	Status anginosus
G33z100	Stenocardia
G33z200	Syncope anginosa
G33z300	Angina on effort
G33z400	Ischaemic chest pain
G33z500	Post infarct angina

G33z600	New onset angina
G33z700	Stable angina
G33zz00	Angina pectoris NOS
G34..00	Other chronic ischaemic heart disease
G349900	Chr. ischaemic heart dis. NOS
G340.00	Coronary atherosclerosis
G340100	Triple vessel disease of the heart
G340200	Coronary artery disease
G340000	Single coronary vessel disease
G340100	Double coronary vessel disease
G342.00	Atherosclerotic cardiovascular disease
G343.00	Ischaemic cardiomyopathy
G344.00	Silent myocardial ischaemia
G34y.00	Other specified chronic ischaemic heart disease
G34y000	Chronic coronary insufficiency
G34y100	Chronic myocardial ischaemia
G34yz00	Other specified chronic ischaemic heart disease NOS
G34z.00	Other chronic ischaemic heart disease NOS
G34z000	Asymptomatic coronary heart disease
G35..00	Subsequent myocardial infarction
G350.00	Subsequent myocardial infarction of anterior wall
G351.00	Subsequent myocardial infarction of inferior wall
G353.00	Subsequent myocardial infarction of other sites
G35X.00	Subsequent myocardial infarction of unspecified site
G36..00	Certain current complication follow acute myocardial infarct
G360.00	Haemopericardium/current comp follow acute myocardial infarct
G361.00	Atrial septal defect/curr comp follow acute myocardial infarct
G362.00	Ventricular septal defect/curr comp follow acute myocardial infarction
G363.00	Ruptur cardiac wall w'out haemopericard/cur comp follow ac MI
G364.00	Ruptur chordae tendinae/curr comp follow acute myocardial infarct
G365.00	Rupture papillary muscle/curr comp follow acute myocardial infarct
G366.00	Thrombosis atrium, auric append&vent/curr comp follow acute MI
G38..00	Postoperative myocardial infarction
G380.00	Postoperative transmural myocardial infarction anterior wall
G381.00	Postoperative transmural myocardial infarction inferior wall
G382.00	Postoperative transmural myocardial infarction other sites
G383.00	Postoperative transmural myocardial infarction unspec site
G384.00	Postoperative subendocardial myocardial infarction
G38z.00	Postoperative myocardial infarction, unspecified
G3y..00	Other specified ischaemic heart disease
G3z..00	Ischaemic heart disease NOS
G501.00	Post infarction pericarditis
Gyu3400	[X]Acute transmural myocardial infarction of unspecif site
F423600	Amaurosis fugax
Fyu5500	[X]Other transnt cerebral ischaemic attacks+related syndromes

G63y000	Cerebral infarct due to thrombosis of precerebral arteries
G63y100	Cerebral infarction due to embolism of precerebral arteries
G64..00	Cerebral arterial occlusion
G641.00	CVA - cerebral artery occlusion
G642.00	Infarction - cerebral
G643.00	Stroke due to cerebral arterial occlusion
G640.00	Cerebral thrombosis
G640000	Cerebral infarction due to thrombosis of cerebral arteries
G641.00	Cerebral embolism
G641100	Cerebral embolus
G641000	Cerebral infarction due to embolism of cerebral arteries
G64z.00	Cerebral infarction NOS
G64z100	Brainstem infarction NOS
G64z200	Cerebellar infarction
G64z990	Cerebral A. occlusion NOS
G64z000	Brainstem infarction
G64z100	Wallenberg syndrome
G64z110	Lateral medullary syndrome
G64z200	Left sided cerebral infarction
G64z300	Right sided cerebral infarction
G64z400	Infarction of basal ganglia
G65..00	Transient cerebral ischaemia
G651.00	Drop attack
G652.00	Transient ischaemic attack
G653.00	Vertebro-basilar insufficiency
G659900	Transient Ischaemic Attacks
G650.00	Basilar artery syndrome
G650100	Insufficiency - basilar artery
G652.00	Subclavian steal syndrome
G653.00	Carotid artery syndrome hemispheric
G654.00	Multiple and bilateral precerebral artery syndromes
G656.00	Vertebrobasilar insufficiency
G65y.00	Other transient cerebral ischaemia
G65z.00	Transient cerebral ischaemia NOS
G65z990	Transient Ischaemic Attacks
G65z000	Impending cerebral ischaemia
G65z100	Intermittent cerebral ischaemia
G65zz00	Transient cerebral ischaemia NOS
G66..00	Stroke and cerebrovascular accident unspecified
G661.00	CVA unspecified
G662.00	Stroke unspecified
G663.00	CVA - Cerebrovascular accident unspecified
G669800	Stroke/CVA - undefined
G669900	Stroke
G667.00	Left sided CVA

G668.00	Right sided CVA
G676000	Cereb infarct due cerebral venous thrombosis, nonpyogenic
G6W..00	Cereb infarct due unspcf occlus/stenos precerebr arteries
G6X..00	Cerebrl infarctn due/unspcf occlusn or sten/cerebrl artrs
Gyu6300	[X]Cerebrl infarctn due/unspcf occlusn or sten/cerebrl artrs
Gyu6400	[X]Other cerebral infarction
Gyu6500	[X]Occlusion and stenosis of other precerebral arteries
Gyu6600	[X]Occlusion and stenosis of other cerebral arteries
ZV12D00	[V]Personal history of transient ischaemic attack

ICD10 code for HES and ONS data	
ICD10-code	description
G45	transient ischaemic attack and related syndromes
G45.0	transient ischaemic attack and related syndromes
G45.1	transient ischaemic attack and related syndromes
G45.2	transient ischaemic attack and related syndromes
G45.3	transient ischaemic attack and related syndromes
G45.4	transient ischaemic attack and related syndromes
G45.8	transient ischaemic attack and related syndromes
G45.9	transient ischaemic attack and related syndromes
I20	angina pectoris
I20.0	angina pectoris
I20.1	angina pectoris
I20.8	angina pectoris
I20.9	angina pectoris
I21	acute myocardial infarction
I21.0	acute myocardial infarction
I21.1	acute myocardial infarction
I21.2	acute myocardial infarction
I21.3	acute myocardial infarction
I21.4	acute myocardial infarction
I21.9	acute myocardial infarction
I22	subsequent myocardial infarction
I22.0	subsequent myocardial infarction
I22.1	subsequent myocardial infarction
I22.8	subsequent myocardial infarction
I22.9	subsequent myocardial infarction
I23	complications after myocardial infarction
I23.0	complications after myocardial infarction
I23.1	complications after myocardial infarction

I23.2	complications after myocardial infarction
I23.3	complications after myocardial infarction
I23.4	complications after myocardial infarction
I23.5	complications after myocardial infarction
I23.6	complications after myocardial infarction
I23.8	complications after myocardial infarction
I24	other acute ischaemic heart disease
I24.0	other acute ischaemic heart disease
I24.1	other acute ischaemic heart disease
I24.8	other acute ischaemic heart disease
I24.9	other acute ischaemic heart disease
I25	chronic ischaemic heart disease
I25.0	chronic ischaemic heart disease
I25.1	chronic ischaemic heart disease
I25.2	chronic ischaemic heart disease
I25.3	chronic ischaemic heart disease
I25.4	chronic ischaemic heart disease
I25.5	chronic ischaemic heart disease
I25.6	chronic ischaemic heart disease
I25.8	chronic ischaemic heart disease
I25.9	chronic ischaemic heart disease
I63	cerebral infarction
I63.0	cerebral infarction
I63.1	cerebral infarction
I63.2	cerebral infarction
I63.3	cerebral infarction
I63.4	cerebral infarction
I63.5	cerebral infarction
I63.6	cerebral infarction
I63.8	cerebral infarction
I63.9	cerebral infarction
I64	stroke not specified as haemorrhage or infarction

Supplementary Appendix 3: Summarising repeated measures of risk predictors using multivariate mixed-effects linear regression models

To optimise the use of repeated measurements of risk predictors in electronic health records to predict future CVD risk, we used sliding landmark approach as described in our previous study.[6] A landmark age is a reference point at which we use risk predictor values collected prior to that age and from which to predict future risk.[6] Participants contributed to the models if they have 1) registered with a general practice at the landmark age, 2) no CVD diagnoses prior to the landmark age.

Repeat measurements of systolic blood pressure (SBP), total cholesterol, high-density lipoprotein (HDL) cholesterol, and glycated haemoglobin (HbA_{1c}) were first summarised using sex-specific multivariate mixed models [7] at each landmark age under the landmark approach.

The following biologically implausible values were set to missing: SBP >250 mmHg or <60 mmHg; total cholesterol >20 mmol/L or <1.75mmol/L; HDL cholesterol >3.1 mmol/L or <0.3 mmol/L; HbA_{1c} <2.5% or > 25%.[8–10]

Let SBP_{ij}, $Total\_cholesterol_{ij}$, $HDL\_cholesterol_{ij}$, $HbA1c_{ij,}$ BP_med_{ij} and $Statin_{ij}$ denote all the repeat measurements of systolic blood pressure, total cholesterol, HDL cholesterol, HbA_{1c}, indication of blood pressure-lowering medication, indication of statin initiation, and indication of diabetes medication for individual i recorded at measurement j. For males and females separately, for each landmark age La = 40, 41, 42, ...,85, we fit a multivariate mixed-effect model with a correlated covariance structure:

$$SBP_{ij} = \alpha_1 + (\beta_1 + \nu_{1i}) \times t_{ij} + \gamma * BP\_med_{ij} + u_{1i} + \varepsilon_{1ij}$$

$$Total\_cholesterol_{ij} = \alpha_2 + (\beta_2 + \nu_{2i}) \times t_{ij} + \delta * Statin_{ij} + u_{2i} + \varepsilon_{2ij}$$

$$HDL\_cholesterol_{ij} = \alpha_3 + (\beta_3 + \nu_{3i}) \times t_{ij} + u_{3i} + \varepsilon_{3ij}$$

$$HbA1c_{ij} = \alpha_4 + (\beta_4 + \nu_{4i}) \times t_{ij} + \lambda * DM\_med_{ij} + u_{4i} + \varepsilon_{4ij}$$

where $$\begin{bmatrix} u_{1i} \\ u_{2i} \\ u_{3i} \\ u_{4i} \\ \nu_{1i} \\ \nu_{2i} \\ \nu_{3i} \\ \nu_{4i} \end{bmatrix} \sim multivariate\ normal \left(\begin{bmatrix} 0 \\ 0 \\ 0 \\ 0 \\ 0 \\ 0 \\ 0 \\ 0 \end{bmatrix}, \begin{bmatrix} \sigma_1^2 & \sigma_{12} & \sigma_{13} & \sigma_{14} & \sigma_{15} & \sigma_{16} & \sigma_{17} & \sigma_{18} \\ \sigma_{12} & \sigma_2^2 & \sigma_{23} & \sigma_{24} & \sigma_{25} & \sigma_{26} & \sigma_{27} & \sigma_{28} \\ \sigma_{13} & \sigma_{23} & \sigma_3^2 & \sigma_{34} & \sigma_{35} & \sigma_{36} & \sigma_{37} & \sigma_{38} \\ \sigma_{14} & \sigma_{24} & \sigma_{34} & \sigma_4^2 & \sigma_{45} & \sigma_{46} & \sigma_{47} & \sigma_{48} \\ \sigma_{15} & \sigma_{25} & \sigma_{35} & \sigma_{45} & \sigma_5^2 & \sigma_{56} & \sigma_{57} & \sigma_{58} \\ \sigma_{16} & \sigma_{26} & \sigma_{36} & \sigma_{46} & \sigma_{56} & \sigma_6^2 & \sigma_{67} & \sigma_{68} \\ \sigma_{17} & \sigma_{27} & \sigma_{37} & \sigma_{47} & \sigma_{57} & \sigma_{67} & \sigma_7^2 & \sigma_{78} \\ \sigma_{18} & \sigma_{28} & \sigma_{38} & \sigma_{48} & \sigma_{58} & \sigma_{68} & \sigma_{78} & \sigma_8^2 \end{bmatrix} \right),$$

and $$\begin{bmatrix} \varepsilon_{1i} \\ \varepsilon_{2i} \\ \varepsilon_{3i} \\ \varepsilon_{4i} \end{bmatrix} \sim multivariate\ normal \left(\begin{bmatrix} 0 \\ 0 \\ 0 \\ 0 \end{bmatrix}, \begin{bmatrix} \sigma_{e1}^2 & 0 & 0 & 0 \\ 0 & \sigma_{e2}^2 & 0 & 0 \\ 0 & 0 & \sigma_{e3}^2 & 0 \\ 0 & 0 & 0 & \sigma_{e4}^2 \end{bmatrix} \right)$$

Here α_1, α_2, α_3 and α_4 represent fixed intercepts for each risk predictor, β_1, β_2, β_3 and β_4 represent fixed slopes for each risk predictor, γ represents an adjustment factor in systolic blood pressure levels for those with an indication of blood pressure-lowering medication, δ represents an adjustment factor in total cholesterol for those with an indication of statin medication, and λ

represents an adjustment factor in HbA_{1c} for those with an indication of diabetes medication treatment.

Terms u_{1i}, u_{2i}, u_{3i} and u_{4i} represent random intercepts; v_{1i}, v_{2i}, v_{3i} and v_{4i}represent random slopes for each risk predictor and are correlated between risk predictors. These random intercepts and slopes are interpreted as the difference in the average level of the predictor for this individual compared to the population average level.

Finally, ε_{1ij}, ε_{2ij}, ε_{3ij} and ε_{4ij} represent uncorrelated residual errors for each risk predictor.

This model allows incomplete records of the risk predictors and includes all individuals with at least one measurement from at least one risk predictor.

In our previous work,[6] we restricted the model derivation to repeat measurements recorded **before** the landmark age, i.e. $j \leq La$. However, we found slight improvements in sensitivity analyses when we used all available repeated measurements recorded **before** and **after** the landmark age, due to extra precision on parameter estimates. We accept a limitation is that it ignores informative censoring of individuals due to death or CVD events, however, our previous work shows informative censoring has little effect on the long-term usual levels of the included risk predictors.

Best linear unbiased predictors (BLUPS)[11] are estimated for each risk predictor for the random intercepts $\hat{u}_{1i}$, $\hat{u}_{2i}$, $\hat{u}_{3i}$ and $\hat{u}_{4i}$ and random slopes using observed data for $j \leq La$.[12] Note the restriction to only repeat measurements before the landmark age is important here, as the prediction model is intended for use in clinical practice where only past data will be available. The BLUPs are estimated as the mean of the empirical Bayes posterior distribution of the random intercepts and slopes conditional on observed risk predictor measurements. Using the properties of multivariate normal distributions, this is also a multivariate normal distribution, and an exact formula for the mean can be calculated.[13]

Specifically, for individual i

$$\begin{bmatrix} u_{1i} \\ u_{2i} \\ u_{3i} \\ u_{4i} \\ v_{1i} \\ v_{2i} \\ v_{3i} \\ v_{4i} \end{bmatrix} = GZ^T(ZGZ^T + \sum)^{-1}(Y - X\beta)$$

Here Y is the vector of risk predictor observations, $X\beta$ is the linear prediction from the fitted model, G is the covariance matrix of the random effects = $\begin{bmatrix} \sigma_1^2 & \sigma_{12} & \sigma_{13} & \sigma_{14} & \sigma_{15} & \sigma_{16} & \sigma_{17} & \sigma_{18} \\ \sigma_{12} & \sigma_2^2 & \sigma_{23} & \sigma_{24} & \sigma_{25} & \sigma_{26} & \sigma_{27} & \sigma_{28} \\ \sigma_{13} & \sigma_{23} & \sigma_3^2 & \sigma_{34} & \sigma_{35} & \sigma_{36} & \sigma_{37} & \sigma_{38} \\ \sigma_{14} & \sigma_{24} & \sigma_{34} & \sigma_4^2 & \sigma_{45} & \sigma_{46} & \sigma_{47} & \sigma_{48} \\ \sigma_{15} & \sigma_{25} & \sigma_{35} & \sigma_{45} & \sigma_5^2 & \sigma_{56} & \sigma_{57} & \sigma_{58} \\ \sigma_{16} & \sigma_{26} & \sigma_{36} & \sigma_{46} & \sigma_{56} & \sigma_6^2 & \sigma_{67} & \sigma_{68} \\ \sigma_{17} & \sigma_{27} & \sigma_{37} & \sigma_{47} & \sigma_{57} & \sigma_{67} & \sigma_7^2 & \sigma_{78} \\ \sigma_{18} & \sigma_{28} & \sigma_{38} & \sigma_{48} & \sigma_{58} & \sigma_{68} & \sigma_{78} & \sigma_8^2 \end{bmatrix}$, Z is the design matrix which selects the corresponding random effect for each risk predictor, Z^T is the

matrix transpose of Z and $\sum$ is a diagonal matrix containing the corresponding residual variance for each risk predictor. Importantly, due to the correlations structure between the random intercepts and slopes, BLUPS can be estimated for all individuals with at least one repeat measurement for at least one risk predictor.

After the estimation of the predictor values in the above step, in a 2/3 derivation dataset, sex-specific “super landmark” Cox models[14] using the stacked data across all landmark ages with robust standard errors were used to derive the 10-year CVD risk prediction model. Continuous predictors were standardized using sex-specific means and standard deviations and entered as linear terms. In addition to the predictors, individual-level slopes and standard deviation terms defined above, we included landmark age, landmark age-squared and landmark age interaction terms with SBP, total cholesterol, HDL cholesterol, HbA1c, and smoking status. The proportional hazards assumption was tested by examining the Schoenfeld residuals, and we did not observe a clear indication of a violation of the assumption in our models. Hazard ratios from the Cox model were then applied to the 1/3 validation dataset to estimate 10-year CVD risk.

Supplementary Appendix 4: Summary of metrics used for model predictive performance assessment

We randomly split our data by practice to a 2/3 derivation dataset (263 practices with 53,292 individuals) and a 1/3 validation dataset (132 practices with 30,618 individuals). Model predictive performance were examined in the validation dataset.

Although bootstrap-based internal validation is considered the most efficient method for internal validation, for feasibility, we chose the split-sample validation at the practical level, which also offers some degree of independence between the split samples since practices are heterogeneous, in terms of data quality, geography/socio-economic factors.[5] Due to the large number of individuals and endpoints per variable of >800, this is unlikely to lead to biased results.[15,16]

Aspect	Metric	Description
Overall performance	Brier score	Squared differences between observed 10-year CVD outcomes and predicted risk.[17]<br>Lower values indicate better accuracy.
Discrimination	Harrell's C-index	A rank-order measure to quantify the discriminative ability to rank individuals according to their predicted and observed risk of CVD.[18,19]<br>Takes values between 0.5 and 1, where 1 means perfect discrimination and 0.5 means by chance alone.
Calibration	Calibration slope	By fitting the Cox survival model with the linear predictor from the prognostic model as the only explanatory variable in the model, the regression coefficient of the linear predictor is the calibration slope.[19,20] Values close to 1 indicate better calibration.
Reclassification	Prospective form of NRI (continuous NRI)	The category-free version of NRI which can include individuals with censored events. It is a measure of the event rate increase among those who are reclassified upwards and the event rate decrease among those who are reclassified downwards.[21]

Supplementary Appendix 5: TRIPOD checklist [22]

Section/Topic	Item	Checklist Item	Page
**Title and abstract**			
Title	1	Identify the study as developing and/or validating a multivariable prediction model, the target population, and the outcome to be predicted.	cover
Abstract	2	Provide a summary of objectives, study design, setting, participants, sample size, predictors, outcome, statistical analysis, results, and conclusions.	3
**Introduction**			
Background and objectives	3a	Explain the medical context (including whether diagnostic or prognostic) and rationale for developing or validating the multivariable prediction model, including references to existing models.	6
	3b	Specify the objectives, including whether the study describes the development or validation of the model or both.	6,7
**Methods**			
Source of data	4a	Describe the study design or source of data (e.g., randomized trial, cohort, or registry data), separately for the development and validation data sets, if applicable.	7,8
	4b	Specify the key study dates, including start of accrual; end of accrual; and, if applicable, end of follow-up.	7,8
Participants	5a	Specify key elements of the study setting (e.g., primary care, secondary care, general population) including number and location of centres.	7,8
	5b	Describe eligibility criteria for participants.	8, Supplementary Figure 1
	5c	Give details of treatments received, if relevant.	NA
Outcome	6a	Clearly define the outcome that is predicted by the prediction model, including how and when assessed.	8, Supplementary Appendix 2
	6b	Report any actions to blind assessment of the outcome to be predicted.	8, Supplementary Appendix 2
Predictors	7a	Clearly define all predictors used in developing or validating the multivariable prediction model, including how and when they were measured.	9, Supplementary Appendix 1
	7b	Report any actions to blind assessment of predictors for the outcome and other predictors.	9
Sample size	8	Explain how the study size was arrived at.	8, Supplementary Figure 1
Missing data	9	Describe how missing data were handled (e.g., complete-case analysis, single imputation, multiple imputation) with details of any imputation method.	8, 11
Statistical analysis methods	10a	Describe how predictors were handled in the analyses.	9,10, Supplementary Appendix 3
	10b	Specify type of model, all model-building procedures (including any predictor selection), and method for internal validation.	9,10, Supplementary Appendix 3

	10d	Specify all measures used to assess model performance and, if relevant, to compare multiple models.	10,11, Supplementary Appendix 4
Risk groups	11	Provide details on how risk groups were created, if done.	NA
**Results**			
Participants	13a	Describe the flow of participants through the study, including the number of participants with and without the outcome and, if applicable, a summary of the follow-up time. A diagram may be helpful.	11, Table 1
	13b	Describe the characteristics of the participants (basic demographics, clinical features, available predictors), including the number of participants with missing data for predictors and outcome.	11,12, Supplementary Table 1, Supplementary Figures 5 and 6
Model development	14a	Specify the number of participants and outcome events in each analysis.	11, Supplementary Figures 3 and 4
	14b	If done, report the unadjusted association between each candidate predictor and outcome.	NA
Model specification	15a	Present the full prediction model to allow predictions for individuals (i.e., all regression coefficients, and model intercept or baseline survival at a given time point).	12, Supplementary Tables 2 and 3
	15b	Explain how to the use the prediction model.	12
Model performance	16	Report performance measures (with CIs) for the prediction model.	12-14, Table 2, Figure 2, Supplementary Tables 7-12, Supplementary Figures 8-17
**Discussion**			
Limitations	18	Discuss any limitations of the study (such as nonrepresentative sample, few events per predictor, missing data).	17-18
Interpretation	19b	Give an overall interpretation of the results, considering objectives, limitations, and results from similar studies, and other relevant evidence.	14-17
Implications	20	Discuss the potential clinical use of the model and implications for future research.	16-18
**Other information**			
Supplementary information	21	Provide information about the availability of supplementary resources, such as study protocol, Web calculator, and data sets.	Supplementary Appendices
Funding	22	Give the source of funding and the role of the funders for the present study.	24,25

Supplementary Appendix 6: Supplementary tables and figures

Supplementary Table S1. Comparison of characteristics of 83,910 participants with type 2 diabetes included in the current study* and 75,820 participants with type 2 diabetes but with missing predictor measurements†

Characteristics	People with complete data on risk predictors (n = 83,910)	People with missing data on risk predictors (n = 75,820)
Age at study entry, mean (SD), years	58.5 (12.2)	59.2 (13.0)
Men, n (%)	51,106 (60.9)	40,689 (53.7)
Ethnicity, n (%)		
White	29,644 (35.3)	20,516 (27.1)
Asian	2,380 (2.8)	6,395 (8.4)
Black	1,491 (1.8)	3,252 (4.3)
Mixed	312 (0.4)	505 (0.7)
Other	670 (0.8)	1,154 (1.5)
Unspecified/missing	49,413 (58.9)	43,980 (58.0)
Incidence of CVD, rate (95% CI), 1,000 person-years	17.5 (17.2, 17.8)	21.6 (21.2, 22.0)

Abbreviations: CI, confidence interval; CVD, cardiovascular disease.

* Included 83,910 individuals from Clinical Practice Research Datalink, Hospital Episode Statistics, and the Office for National Statistics, England, United Kingdom, 2004-2017, aged 40-85 years, without prevalent CVD before study entry, with confirmed Type 2 diabetes before incident CVD events (if any) and/or study exit, and complete data on measurements of SBP, total cholesterol, HDL cholesterol, HbA_{1c}, and smoking status between their study entry and study exit dates.

† The 75,820 participants not included in the study were those with type 2 diabetes but have no detected measurements for complete risk predictors of SBP, total cholesterol, HDL cholesterol, HbA1c, and smoking status before CVD events (if any) and/or study exit.

Supplementary Table S2. Hazard ratios* of risk predictors and interaction terms included in the Cox model for cardiovascular disease risk derived from different risk prediction models for **men** with type 2 diabetes in the derivation dataset, Clinical Practice Research Datalink, Hospital Episode Statistics, and the Office for National Statistics, England, United Kingdom, 2004-2017

Risk predictor	Last observed value model †	Mean value model ‡	Estimated current value model §
	HR (95% CI)	HR (95% CI)	HR (95% CI)
Age	1.04 (1.02, 1.06)	1.04 (1.02, 1.06)	1.05 (1.03, 1.07)
Age squared	1.00 (1.00, 1.00)	1.00 (1.00, 1.00)	1.00 (1.00, 1.00)
Duration of diabetes	1.02 (1.02, 1.03)	1.03 (1.02, 1.03)	1.02 (1.02, 1.03)
SBP	1.32 (1.16, 1.51)	1.42 (1.21, 1.67)	1.37 (1.14, 1.65)
Total cholesterol	1.57 (1.40, 1.76)	1.63 (1.42, 1.88)	2.23 (1.90, 2.61)
HDL cholesterol	0.57 (0.52, 0.64)	0.56 (0.50, 0.63)	0.51 (0.45, 0.58)
HbA_{1c}	1.09 (0.96, 1.23)	1.04 (0.90, 1.21)	1.10 (0.93, 1.30)
Current smoking	2.36 (1.87, 2.98)	2.38 (1.88, 3.01)	2.33 (1.84, 2.95)
History of atrial fibrillation	1.92 (1.82, 2.03)	1.94 (1.83, 2.05)	1.94 (1.84, 2.05)
Blood pressure-lowering medication use	1.35 (1.30, 1.41)	1.32 (1.26, 1.38)	1.33 (1.27, 1.39)
Ethnicity			
White	Ref.	Ref.	Ref.
Mixed	1.11 (0.83, 1.49)	1.15 (0.86, 1.54)	1.13 (0.84, 1.52)
Asian	1.34 (1.23, 1.46)	1.35 (1.24, 1.47)	1.36 (1.25, 1.48)
Black or Black British	0.97 (0.86, 1.11)	0.99 (0.88, 1.13)	0.98 (0.86, 1.12)
Other	1.21 (1.01, 1.46)	1.23 (1.03, 1.48)	1.22 (1.01, 1.47)
Unspecified	0.99 (0.94, 1.04)	0.99 (0.94, 1.03)	0.99 (0.94, 1.04)
Missing	1.05 (1.01, 1.08)	1.04 (1.01, 1.07)	1.04 (1.01, 1.07)
Age×SBP	1.00 (1.00, 1.00)	1.00 (0.99, 1.00)	1.00 (0.99, 1.00)
Age×Total cholesterol	1.00 (0.99, 1.00)	1.00 (0.99, 1.00)	0.99 (0.99, 0.99)
Age×HDL cholesterol	1.01 (1.01, 1.01)	1.01 (1.01, 1.01)	1.01 (1.01, 1.01)
Age×HbA_{1c}	1.00 (1.00, 1.00)	1.00 (1.00, 1.00)	1.00 (1.00, 1.00)
Age×Current smoking	0.99 (0.99, 0.99)	0.99 (0.99, 0.99)	0.99 (0.99, 0.99)

Abbreviations: CI, confidence interval; HDL, high-density lipoprotein; HbA_{1c}, glycated haemoglobin; SBP, systolic blood pressure.

* Hazard ratios are given pre standard deviation increase for SBP, total cholesterol, HDL cholesterol, and HbA_{1c}; and per year increase in duration of type 2 diabetes.

† Last observed value: Sex-specific Cox regression model that used estimated risk factor values of SBP, total cholesterol, HDL cholesterol, and HbA_{1c} from **last observed values** prior to the landmark age, together with landmark age, landmark age squared, ethnicity, duration of diabetes, smoking status, blood pressure-lowering medication use, and atrial fibrillation status, plus landmark age interaction terms with SBP, total cholesterol, HDL cholesterol, HbA_{1c}, and smoking status.

‡ Mean value: Sex-specific Cox regression model that used estimated risk factor values of SBP, total cholesterol, HDL cholesterol, and HbA_{1c} from **cumulative means** of the prior repeated measures, together with other risk factors and interaction terms as same as the last observed value model.

§ Estimated current value: Sex-specific Cox regression model that used **estimated current risk factor values** at the landmark age of SBP, total cholesterol, HDL cholesterol, and HbA_{1c} from **multivariate mixed-effects linear regression models** of the prior repeated measures, together with other risk factors and interaction terms as same as the last observed value model.

Supplementary Table S3. Hazard ratios* of risk predictors and interaction terms included in the Cox model for cardiovascular disease risk derived from different risk prediction models for **women** with type 2 diabetes in the derivation dataset, Clinical Practice Research Datalink, Hospital Episode Statistics, and the Office for National Statistics, England, United Kingdom, 2004-2017

**Risk predictor**	**Last observed value model †**	**Mean value model ‡**	**Estimated current value model §**
	**HR (95% CI)**	**HR (95% CI)**	**HR (95% CI)**
Age	1.02 (0.99, 1.05)	1.02 (0.99, 1.04)	1.03 (1.00, 1.06)
Age squared	1.00 (1.00, 1.00)	1.00 (1.00, 1.00)	1.00 (1.00, 1.00)
Duration of diabetes	1.03 (1.02, 1.03)	1.03 (1.03, 1.03)	1.03 (1.03, 1.03)
SBP	1.23 (1.04, 1.45)	1.01 (0.81, 1.26)	1.11 (0.87, 1.42)
Total cholesterol	1.27 (1.09, 1.48)	1.52 (1.27, 1.82)	1.48 (1.21, 1.82)
HDL cholesterol	0.74 (0.64, 0.87)	0.77 (0.65, 0.92)	0.71 (0.60, 0.86)
HbA_{1c}	1.83 (1.58, 2.12)	1.96 (1.65, 2.33)	2.15 (1.78, 2.59)
Current smoking	1.80 (1.32, 2.44)	1.71 (1.26, 2.33)	1.72 (1.27, 2.34)
History of atrial fibrillation	2.28 (2.11, 2.47)	2.30 (2.12, 2.48)	2.31 (2.13, 2.50)
Blood pressure-lowering medication use	1.56 (1.46, 1.67)	1.54 (1.43, 1.65)	1.54 (1.44, 1.65)
Ethnicity			
White	Ref.	Ref.	Ref.
Mixed	0.67 (0.45, 1.01)	0.69 (0.46, 1.03)	0.67 (0.45, 1.00)
Asian	1.13 (0.93, 1.37)	1.11 (0.92, 1.35)	1.12 (0.93, 1.36)
Black or Black British	0.71 (0.57, 0.89)	0.69 (0.55, 0.86)	0.70 (0.56, 0.88)
Other	0.72 (0.46, 1.15)	0.73 (0.46, 1.17)	0.74 (0.47, 1.18)
Unspecified	1.05 (0.98, 1.12)	1.05 (0.98, 1.12)	1.05 (0.99, 1.12)
Missing	1.06 (1.02, 1.11)	1.06 (1.01, 1.10)	1.06 (1.01, 1.10)
Age×SBP	1.00 (1.00, 1.00)	1.00 (1.00, 1.00)	1.00 (1.00, 1.00)
Age×Total cholesterol	1.00 (1.00, 1.00)	1.00 (0.99, 1.00)	1.00 (0.99, 1.00)
Age×HDL cholesterol	1.00 (1.00, 1.01)	1.00 (1.00, 1.00)	1.00 (1.00, 1.01)
Age×HbA_{1c}	0.99 (0.99, 1.00)	0.99 (0.99, 1.00)	0.99 (0.99, 0.99)
Age×Current smoking	1.00 (0.99, 1.00)	1.00 (0.99, 1.00)	1.00 (0.99, 1.00)

Abbreviations: CI, confidence interval; HDL, high-density lipoprotein; HbA_{1c}, glycated haemoglobin; SBP, systolic blood pressure.

* Hazard ratios are given pre standard deviation increase for SBP, total cholesterol, HDL cholesterol, and HbA_{1c}; and per year increase in duration of type 2 diabetes.

† Last observed value: Sex-specific Cox regression model that used estimated risk factor values of SBP, total cholesterol, HDL cholesterol, and HbA_{1c} from **last observed values** prior to the landmark age, together with landmark age, landmark age squared, ethnicity, duration of diabetes, smoking status, blood pressure-lowering medication use, and atrial fibrillation status, plus landmark age interaction terms with SBP, total cholesterol, HDL cholesterol, HbA_{1c}, and smoking status.

‡ Mean value: Sex-specific Cox regression model that used estimated risk factor values of SBP, total cholesterol, HDL cholesterol, and HbA_{1c} from **cumulative means** of the prior repeated measures, together with other risk factors and interaction terms as same as the last observed value model.

§ Estimated current value: Sex-specific Cox regression model that used **estimated current risk factor values** at the landmark age of SBP, total cholesterol, HDL cholesterol, and HbA_{1c} from **multivariate mixed-effects linear regression models** of the prior repeated measures, together with other risk factors and interaction terms as same as the last observed value model.

Supplementary Table S4. Hazard ratios* of risk predictors, interaction terms, plus **standard deviations** included in the Cox model for cardiovascular disease risk derived from different risk prediction models for **men** with type 2 diabetes in the derivation dataset, Clinical Practice Research Datalink, Hospital Episode Statistics, and the Office for National Statistics, England, United Kingdom, 2004-2017

Risk predictor	Last observed value + SD model †	Mean value + SD model ‡	Estimated current value + SD model §
	HR (95% CI)	HR (95% CI)	HR (95% CI)
Age	1.05 (1.03, 1.07)	1.05 (1.03, 1.08)	1.07 (1.04, 1.09)
Age squared	1.00 (1.00, 1.00)	1.00 (1.00, 1.00)	1.00 (1.00, 1.00)
Duration of diabetes	1.02 (1.02, 1.03)	1.02 (1.02, 1.03)	1.02 (1.02, 1.03)
SBP	1.39 (1.21, 1.61)	1.43 (1.19, 1.72)	1.40 (1.14, 1.72)
Standard deviation	1.03 (1.03, 1.03)	1.03 (1.02, 1.03)	1.03 (1.02, 1.03)
Total cholesterol	1.67 (1.46, 1.90)	1.95 (1.65, 2.31)	2.49 (2.08, 2.99)
Standard deviation	0.96 (0.93, 1.00)	0.84 (0.81, 0.88)	0.90 (0.87, 0.94)
HDL cholesterol	0.52 (0.46, 0.59)	0.47 (0.41, 0.54)	0.44 (0.39, 0.51)
Standard deviation	1.95 (1.59, 2.38)	2.54 (2.06, 3.12)	2.22 (1.81, 2.72)
HbA_{1c}	1.12 (0.98, 1.29)	1.11 (0.93, 1.34)	1.13 (0.94, 1.36)
Standard deviation	1.08 (1.05, 1.11)	1.01 (0.98, 1.04)	1.05 (1.02, 1.08)
Current smoking	2.50 (1.92, 3.26)	2.49 (1.91, 3.25)	2.44 (1.87, 3.19)
History of atrial fibrillation	1.85 (1.74, 1.96)	1.84 (1.74, 1.96)	1.86 (1.75, 1.97)
Blood pressure-lowering medication use	1.26 (1.20, 1.32)	1.27 (1.21, 1.33)	1.26 (1.20, 1.32)
Ethnicity			
White	Ref.	Ref.	Ref.
Mixed	1.15 (0.84, 1.59)	1.16 (0.85, 1.60)	1.16 (0.84, 1.60)
Asian	1.28 (1.16, 1.41)	1.28 (1.16, 1.40)	1.30 (1.18, 1.43)
Black or Black British	0.93 (0.81, 1.07)	0.95 (0.83, 1.10)	0.94 (0.82, 1.08)
Other	1.15 (0.94, 1.41)	1.15 (0.93, 1.40)	1.15 (0.94, 1.41)
Unspecified	0.99 (0.94, 1.04)	0.98 (0.94, 1.04)	0.99 (0.94, 1.04)
Missing	1.02 (0.99, 1.06)	1.02 (0.98, 1.05)	1.02 (0.98, 1.06)
Age×SBP	1.00 (0.99, 1.00)	1.00 (0.99, 1.00)	1.00 (0.99, 1.00)
Age×Total cholesterol	1.00 (0.99, 1.00)	0.99 (0.99, 1.00)	0.99 (0.99, 0.99)
Age×HDL cholesterol	1.01 (1.01, 1.01)	1.01 (1.01, 1.01)	1.01 (1.01, 1.01)
Age×HbA_{1c}	1.00 (1.00, 1.00)	1.00 (1.00, 1.00)	1.00 (1.00, 1.00)
Age×Current smoking	0.99 (0.98, 0.99)	0.99 (0.98, 0.99)	0.99 (0.99, 0.99)

Abbreviations: CI, confidence interval; HDL, high-density lipoprotein; HbA_{1c}, glycated haemoglobin; SBP, systolic blood pressure.

* Hazard ratios are given pre standard deviation increase for SBP, total cholesterol, HDL cholesterol, and HbA_{1c}; and per year increase in duration of type 2 diabetes.

† Last observed value + SD model: Sex-specific Cox regression model that used estimated risk predictor values of SBP, total cholesterol, HDL cholesterol, and HbA_{1c} from **last observed values** prior to the landmark age plus individual-level **standard deviations** of SBP, total cholesterol, HDL cholesterol, and HbA_{1c}, together with landmark age, landmark age squared, ethnicity, duration of diabetes, smoking status, blood pressure-lowering medication use, and atrial fibrillation status, plus landmark age interaction terms with SBP, total cholesterol, HDL cholesterol, HbA_{1c}, and smoking status.

‡ Mean value + SD model: Sex-specific Cox regression model that used estimated risk predictor values of SBP, total cholesterol, HDL cholesterol, and HbA_{1c} from cumulative mean (**Mean**) of the historical repeated

measures, plus individual-level **standard deviations** of SBP, total cholesterol, HDL cholesterol, and HbA_{1c}, together with other risk predictors and interaction terms as same as last observed value + SD model.

§ Estimated current value + SD model: Sex-specific Cox regression model that used **estimated current risk predictor values** of SBP, total cholesterol, HDL cholesterol, and HbA_{1c} from multivariate mixed-effects linear regression models of the historical repeated measures, plus individual-level **standard deviations** of SBP, total cholesterol, HDL cholesterol, and HbA_{1c}, together with other risk predictors and interaction terms as same as last observed value + SD model.

Supplementary Table S5. Hazard ratios* of risk predictors, interaction terms, plus **standard deviations** included in the Cox model for cardiovascular disease risk derived from different risk prediction models for **women** with type 2 diabetes in the derivation dataset, Clinical Practice Research Datalink, Hospital Episode Statistics, and the Office for National Statistics, England, United Kingdom, 2004-2017

**Risk predictor**	**Last observed value + SD model †**	**Mean value + SD model ‡**	**Estimated current value + SD model §**
	**HR (95% CI)**	**HR (95% CI)**	**HR (95% CI)**
Age	1.02 (0.99, 1.05)	1.03 (1.00, 1.06)	1.04 (1.01, 1.07)
Age squared	1.00 (1.00, 1.00)	1.00 (1.00, 1.00)	1.00 (1.00, 1.00)
Duration of diabetes	1.03 (1.02, 1.03)	1.03 (1.02, 1.03)	1.03 (1.02, 1.03)
SBP	1.25 (1.04, 1.50)	0.99 (0.77, 1.27)	1.10 (0.84, 1.44)
Standard deviation	1.02 (1.01, 1.02)	1.02 (1.01, 1.02)	1.02 (1.01, 1.02)
Total cholesterol	1.33 (1.12, 1.59)	1.74 (1.40, 2.15)	1.57 (1.25, 1.96)
Standard deviation	0.92 (0.88, 0.97)	0.84 (0.80, 0.89)	0.89 (0.84, 0.93)
HDL cholesterol	0.67 (0.57, 0.80)	0.67 (0.55, 0.82)	0.64 (0.52, 0.77)
Standard deviation	1.89 (1.46, 2.45)	2.20 (1.69, 2.87)	2.07 (1.59, 2.69)
HbA_{1c}	1.84 (1.56, 2.18)	2.35 (1.92, 2.88)	2.27 (1.85, 2.79)
Standard deviation	1.13 (1.09, 1.17)	1.05 (1.01, 1.09)	1.09 (1.05, 1.14)
Current smoking	1.71 (1.22, 2.41)	1.56 (1.11, 2.19)	1.62 (1.15, 2.28)
History of atrial fibrillation	2.15 (1.97, 2.34)	2.17 (1.99, 2.37)	2.18 (2.00, 2.38)
Blood pressure-lowering medication use	1.50 (1.39, 1.62)	1.51 (1.39, 1.63)	1.50 (1.38, 1.62)
Ethnicity			
White	Ref.	Ref.	Ref.
Mixed	0.69 (0.45, 1.05)	0.71 (0.46, 1.08)	0.69 (0.45, 1.05)
Asian	1.09 (0.88, 1.34)	1.09 (0.88, 1.35)	1.10 (0.89, 1.35)
Black or Black British	0.68 (0.53, 0.87)	0.67 (0.52, 0.85)	0.68 (0.53, 0.87)
Other	0.73 (0.45, 1.19)	0.72 (0.44, 1.18)	0.74 (0.46, 1.21)
Unspecified	1.03 (0.97, 1.11)	1.03 (0.96, 1.11)	1.04 (0.97, 1.11)
Missing	1.06 (1.01, 1.11)	1.05 (1.01, 1.11)	1.05 (1.01, 1.11)
Age×SBP	1.00 (1.00, 1.00)	1.00 (1.00, 1.01)	1.00 (1.00, 1.00)
Age×Total cholesterol	1.00 (1.00, 1.00)	0.99 (0.99, 1.00)	1.00 (0.99, 1.00)
Age×HDL cholesterol	1.00 (1.00, 1.01)	1.00 (1.00, 1.01)	1.00 (1.00, 1.01)
Age×HbA_{1c}	0.99 (0.99, 1.00)	0.99 (0.99, 0.99)	0.99 (0.99, 0.99)
Age×Current smoking	1.00 (0.99, 1.00)	1.00 (0.99, 1.00)	1.00 (0.99, 1.00)

Abbreviations: CI, confidence interval; HDL, high-density lipoprotein; HbA_{1c}, glycated haemoglobin; SBP, systolic blood pressure.

* Hazard ratios are given pre standard deviation increase for SBP, total cholesterol, HDL cholesterol, and HbA_{1c}; and per year increase in duration of type 2 diabetes.

† Last observed value + SD model: Sex-specific Cox regression model that used estimated risk predictor values of SBP, total cholesterol, HDL cholesterol, and HbA_{1c} from **last observed values** prior to the landmark age plus individual-level **standard deviations** of SBP, total cholesterol, HDL cholesterol, and HbA_{1c}, together with landmark age, landmark age squared, ethnicity, duration of diabetes, smoking status, blood pressure-lowering medication use, and atrial fibrillation status, plus landmark age interaction terms with SBP, total cholesterol, HDL cholesterol, HbA_{1c}, and smoking status.

‡ Mean value + SD model: Sex-specific Cox regression model that used estimated risk predictor values of SBP, total cholesterol, HDL cholesterol, and HbA_{1c} from cumulative mean (**Mean**) of the historical repeated

measures, plus individual-level **standard deviations** of SBP, total cholesterol, HDL cholesterol, and HbA_{1c}, together with other risk predictors and interaction terms as same as last observed value + SD model.

§ Estimated current value + SD model: Sex-specific Cox regression model that used **estimated current risk predictor values** of SBP, total cholesterol, HDL cholesterol, and HbA_{1c} from multivariate mixed-effects linear regression models of the historical repeated measures, plus individual-level **standard deviations** of SBP, total cholesterol, HDL cholesterol, and HbA_{1c}, together with other risk predictors and interaction terms as same as last observed value + SD model.

Supplementary Table S6. Hazard ratios* of risk predictors, interaction terms, plus **random slopes** included in the Cox model† for cardiovascular disease risk derived from different risk prediction models for people with type 2 diabetes in the derivation dataset, Clinical Practice Research Datalink, Hospital Episode Statistics, and the Office for National Statistics, England, United Kingdom, 2004-2017

**Risk predictor**	**Men**	**Women**
	**HR (95% CI)**	**HR (95% CI)**
Age	1.06 (1.04, 1.08)	1.04 (1.01, 1.06)
Age squared	1.00 (1.00, 1.00)	1.00 (1.00, 1.00)
Duration of diabetes	1.02 (1.02, 1.03)	1.03 (1.02, 1.03)
SBP	1.36 (1.13, 1.64)	1.08 (0.85, 1.38)
Random slope	0.99 (0.97, 1.00)	0.99 (0.97, 1.01)
Total cholesterol	2.35 (2.00, 2.74)	1.58 (1.29, 1.93)
Random slope	1.12 (0.80, 1.56)	0.98 (0.65, 1.47)
HDL cholesterol	0.48 (0.42, 0.54)	0.68 (0.57, 0.82)
Random slope	3.81 (1.11, 13.07)	1.15 (0.25, 5.30)
HbA_{1c}	1.14 (0.97, 1.35)	2.30 (1.91, 2.77)
Random slope	0.59 (0.51, 0.67)	0.52 (0.44, 0.61)
Current smoking	2.31 (1.83, 2.92)	1.71 (1.26, 2.33)
History of atrial fibrillation	1.95 (1.85, 2.07)	2.31 (2.13, 2.50)
Blood pressure-lowering medication use	1.33 (1.28, 1.39)	1.54 (1.43, 1.65)
Ethnicity		
White	Ref.	Ref.
Mixed	1.13 (0.84, 1.52)	0.67 (0.45, 1.00)
Asian	1.35 (1.24, 1.47)	1.11 (0.91, 1.34)
Black or Black British	0.98 (0.86, 1.11)	0.68 (0.55, 0.86)
Other	1.21 (1.01, 1.46)	0.74 (0.46, 1.18)
Unspecified	0.99 (0.94, 1.03)	1.05 (0.98, 1.12)
Missing	1.04 (1.01, 1.07)	1.06 (1.01, 1.10)
Age×SBP	1.00 (0.99, 1.00)	1.00 (1.00, 1.00)
Age×Total cholesterol	0.99 (0.99, 0.99)	1.00 (0.99, 1.00)
Age×HDL cholesterol	1.01 (1.01, 1.01)	1.00 (1.00, 1.01)
Age×HbA_{1c}	1.00 (1.00, 1.00)	0.99 (0.99, 1.00)
Age×Current smoking	0.99 (0.99, 0.99)	1.00 (0.99, 1.00)

Abbreviations: CI, confidence interval; HDL, high-density lipoprotein; HbA_{1c}, glycated haemoglobin; SBP, systolic blood pressure.

* Hazard ratios are given pre standard deviation increase for SBP, total cholesterol, HDL cholesterol, and HbA_{1c}; and per year increase in duration of type 2 diabetes.

† Sex-specific Cox regression model that used estimated risk predictor values of SBP, total cholesterol, HDL cholesterol, and HbA_{1c} from multivariate mixed-effects linear regression models of the historical repeated measures, plus individual-level **random slope terms** of SBP, total cholesterol, HDL cholesterol, and HbA_{1c}, together with landmark age, landmark age squared, ethnicity, duration of diabetes, smoking status, blood pressure-lowering medication use, and atrial fibrillation status, plus landmark age interaction terms with SBP, total cholesterol, HDL cholesterol, HbA_{1c}, and smoking status.

Supplementary Table S7. C-index and change in C-index with and without well-established predictors for cardiovascular disease risk prediction for people with type 2 diabetes in the validation dataset (N=28,285)[1], Clinical Practice Research Datalink, Hospital Episode Statistics, and the Office for National Statistics, England, United Kingdom, 2004-2017

Model	Overall[2]	
	C-index (95% CI)	Difference (95% CI)
Model with last observed values[3]	0.652 (0.647, 0.656)	Referent
Model with last observed values without SBP[4]	0.645 (0.641, 0.650)	-0.007 (-0.008, -0.005)
Model with last observed values without cholesterol[5]	0.646 (0.641, 0.650)	-0.006 (-0.007, -0.004)
Model with last observed values without HbA_{1c}[6]	0.650 (0.646, 0.654)	-0.002 (-0.001, -0.003)
Model with mean values[7]	0.650 (0.646, 0.655)	Referent
Model with mean values without SBP[8]	0.644 (0.639, 0.648)	-0.006 (-0.007, -0.005)
Model with mean values without cholesterol[9]	0.646 (0.642, 0.651)	-0.004 (-0.005, -0.002)
Model with mean values without HbA_{1c}[10]	0.646 (0.642, 0.651)	-0.004 (-0.005, -0.002)
Model with estimated current values[11]	0.652 (0.648, 0.657)	Referent
Model with estimated current values without SBP[12]	0.646 (0.641, 0.650)	-0.007 (-0.008, -0.005)
Model with estimated current values without cholesterol[13]	0.648 (0.643, 0.653)	-0.004 (-0.006, -0.003)
Model with estimated current values without HbA_{1c}[14]	0.650 (0.645, 0.654)	-0.003 (-0.004, -0.002)

Abbreviations: CI, confidence interval; HDL, high-density lipoprotein; HbA_{1c}, glycated haemoglobin; SBP, systolic blood pressure.

[1] Results shown in this table were calculated among type 2 diabetes individuals with **at least one** measurement for each of systolic blood pressure (SBP), total cholesterol, high-density lipoprotein (HDL) cholesterol, and glycated haemoglobin (HbA_{1c}) prior to each landmark age in the validation dataset from 1/3 study population (N=28,285).

[2] Overall C-index was calculated using combined data of the predicted risk for men and women; predicted risk was estimated from sex-specific Cox models.

[3] Model with last observed values: Sex-specific Cox regression model that used estimated risk factor values of SBP, total cholesterol, HDL cholesterol, and HbA_{1c} from **last observed values** prior to the landmark age, together with landmark age, landmark age squared, ethnicity, duration of diabetes, smoking status, blood pressure-lowering medication use, and atrial fibrillation status, plus landmark age interaction terms with SBP, total cholesterol, HDL cholesterol, HbA_{1c}, and smoking status.

[4] Model with last observed values without SBP: Last observed value model without SBP, blood pressure-lowering medication use, and landmark age interaction terms with SBP.

[5] Model with last observed values without cholesterol: Last observed value model without total cholesterol, HDL cholesterol, and landmark age interaction terms with total cholesterol, HDL cholesterol.

[6] Model with last observed values without HbA_{1c}: Last observed value model without HbA_{1c} and landmark age interaction terms with HbA_{1c}.

[7] Model with mean values: Sex-specific Cox regression model that used estimated risk factor values of SBP, total cholesterol, HDL cholesterol, and HbA_{1c} from **cumulative means** of the prior repeated measures, together with other risk factors and interaction terms as same as last observed value model.

[8] Model with mean values without SBP: Mean model without SBP, blood pressure-lowering medication use, and landmark age interaction terms with SBP.

[9] Model with mean values without cholesterol: Mean model without total cholesterol, HDL cholesterol, and landmark age interaction terms with total cholesterol, HDL cholesterol.

[10] Model with mean values without HbA_{1c}: Mean model without HbA_{1c} and landmark age interaction terms with HbA_{1c}.

[11] Model with estimated current values: Sex-specific Cox regression model that used estimated current risk factor values at the landmark age of SBP, total cholesterol, HDL cholesterol, and HbA_{1c} from **multivariate mixed-effects linear regression models** of the prior repeated measures, together with other risk factors and interaction terms as same as last observed value model.

[12] Model with estimated current values without SBP: Estimated value model without SBP, blood pressure-lowering medication use, and landmark age interaction terms with SBP.

[13] Model with estimated current values without cholesterol: Estimated value model without total cholesterol, HDL cholesterol, and landmark age interaction terms with total cholesterol, HDL cholesterol.

[14] Model with estimated current values without HbA_{1c}: Estimated value model without HbA_{1c} and landmark age interaction terms with HbA_{1c}.

Supplementary Table S8. Brier score for cardiovascular disease risk prediction for people with type 2 diabetes and with at least two measurements in the validation dataset (N=25,512)*, Clinical Practice Research Datalink, Hospital Episode Statistics, and the Office for National Statistics, England, United Kingdom, 2004-2017

Model	Overall		Men		Women	
	**Brier score (95% CI)**	**Difference (95% CI)**	**Brier score (95% CI)**	**Difference (95% CI)**	**Brier score (95% CI)**	**Difference (95% CI)**
Last observed value †	0.352 (0.350, 0.355)	Referent	0.348 (0.345, 0.352)	Referent	0.360 (0.354, 0.367)	Referent
Last observed value + SD‡	0.343 (0.340, 0.345)	-0.010 (-0.010, -0.010)	0.339 (0.335, 0.342)	-0.009 (-0.009, -0.009)	0.350 (0.344, 0.355)	-0.011 (-0.011, -0.011)
Mean value + SD§	0.344 (0.341, 0.346)	-0.009 (-0.009, -0.009)	0.339 (0.336, 0.341)	-0.009 (-0.009, -0.009)	0.351 (0.346, 0.357)	-0.009 (-0.009, -0.009)
Estimated current value + SD model ∣∣	0.344 (0.341, 0.347)	-0.009 (-0.009, -0.009)	0.340 (0.336, 0.342)	-0.009 (-0.009, -0.009)	0.351 (0.346, 0.357)	-0.009 (-0.009, -0.009)

Note: lower Brier score = better accuracy

Abbreviation: CI, confidence interval.

* Results shown in this table were calculated among type 2 diabetes individuals with **at least two measurements** for each of systolic blood pressure (SBP), total cholesterol, high-density lipoprotein (HDL) cholesterol, and glycated haemoglobin (HbA_{1c}) prior to each landmark age in the validation dataset from 1/3 study population (N=25,512).

† Last observed value model: Sex-specific Cox regression model that used estimated risk factor values of SBP, total cholesterol, HDL cholesterol, and HbA_{1c} from **last observed values** prior to the landmark age, together with landmark age, landmark age squared, ethnicity, duration of diabetes, smoking status, blood pressure-lowering medication use, and atrial fibrillation status, plus landmark age interaction terms with SBP, total cholesterol, HDL cholesterol, HbA_{1c}, and smoking status.

‡ Last observed value + SD model: Last observed value model plus individual-level **standard deviations** of SBP, total cholesterol, HDL cholesterol, and HbA_{1c}.

§ Mean value + SD model: Sex-specific Cox regression model that used estimated risk predictor values of SBP, total cholesterol, HDL cholesterol, and HbA_{1c} from cumulative mean (**Mean**) of the historical repeated measures, plus individual-level **standard deviations** of SBP, total cholesterol, HDL cholesterol, and HbA_{1c}, together with other risk predictors and interaction terms as same as the last observed value model.

∣∣ Estimated current value + SD model: Sex-specific Cox regression model that used **estimated current risk predictor values** of SBP, total cholesterol, HDL cholesterol, and HbA_{1c} from **multivariate mixed-effects linear regression model** of the historical repeated measures, plus individual-level **standard deviations** of SBP, total cholesterol, HDL cholesterol, and HbA_{1c}, together with other risk predictors and interaction terms as same as the last observed value model.

Supplementary Table S9. Changes in 10-year cardiovascular disease risk classification* for people with type 2 diabetes in the validation dataset (N=28,285)†, Clinical Practice Research Datalink, Hospital Episode Statistics, and the Office for National Statistics, England, United Kingdom, 2004-2017

Men	NRI	Last observed value model ‡	Mean value model §	Estimated current value model ǁ
Landmark age 40	Non-event NRI (95% CI)	Referent	0.0639 (-0.0466, 0.1744)	0.0110 (-0.0506, 0.0725)
	Event NRI (95% CI)	Referent	-0.2399 (-0.7146, 0.2349)	0.0657 (-0.0819, 0.2134)
	Overall NRI (95% CI)	Referent	-0.1759 (-0.7169, 0.3650)	0.0767 (-0.1147, 0.2681)
Landmark age 50	Non-event NRI (95% CI)	Referent	0.0139 (-0.0705, 0.0983)	0.1907 (0.0754, 0.3061)
	Event NRI (95% CI)	Referent	-0.0187 (-0.1565, 0.1191)	-0.1194 (-0.6330, 0.3942)
	Overall NRI (95% CI)	Referent	-0.0048 (-0.2150, 0.2055)	0.0713 (-0.5007, 0.6433)
Landmark age 60	Non-event NRI (95% CI)	Referent	0.1424 (0.0632, 0.2217)	0.0903 (0.0074, 0.1732)
	Event NRI (95% CI)	Referent	-0.1126 (-0.3767, 0.1515)	-0.1522 (-0.2807, -0.0238)
	Overall NRI (95% CI)	Referent	0.0298 (-0.2890, 0.3485)	-0.0620 (-0.2580, 0.1340)
Landmark age 70	Non-event NRI (95% CI)	Referent	0.1370 (-0.0486, 0.3226)	0.1312 (0.0677, 0.1948)
	Event NRI (95% CI)	Referent	-0.1260 (-0.2861, 0.0341)	-0.0942 (-0.3471, 0.1588)
	Overall NRI (95% CI)	Referent	0.0110 (-0.3178, 0.3398)	0.0371 (-0.2578, 0.3319)
Landmark age 80	Non-event NRI (95% CI)	Referent	0.1479 (0.0909, 0.2050)	0.1423 (-0.0403, 0.3249)
	Event NRI (95% CI)	Referent	0.0625 (-0.0736, 0.1985)	-0.1385 (-0.2953, 0.0184)
	Overall NRI (95% CI)	Referent	0.2104 (0.0363, 0.3845)	0.0038 (-0.3214, 0.3291)
**Women**	**NRI**	**Last observed value model**	**Mean value model**	**Estimated current value model**
Landmark age 40	Non-event NRI (95% CI)	Referent	0.0459 (-0.1004, 0.1922)	0.2215 (0.0668, 0.3762)
	Event NRI (95% CI)	Referent	0.3445 (-0.3610, 1.0499)	0.1913 (-0.5517, 0.9343)
	Overall NRI (95% CI)	Referent	0.3903 (-0.4079, 1.1886)	0.4128 (-0.4349, 1.2606)
Landmark age 50	Non-event NRI (95% CI)	Referent	0.0739 (-0.0076, 0.1553)	0.1273 (0.0442, 0.2104)
	Event NRI (95% CI)	Referent	0.0323 (-0.2704, 0.3351)	-0.0728 (-0.3691, 0.2235)
	Overall NRI (95% CI)	Referent	0.1062 (-0.2453, 0.4577)	0.0545 (-0.2929, 0.4019)
Landmark age 60	Non-event NRI (95% CI)	Referent	0.0620 (-0.0268, 0.1509)	0.0951 (0.0196, 0.1706)
	Event NRI (95% CI)	Referent	0.1091 (-0.1077, 0.3260)	0.0334 (-0.1721, 0.2389)
	Overall NRI (95% CI)	Referent	0.1712 (-0.1083, 0.4506)	0.1285 (-0.1281, 0.3850)
Landmark age 70	Non-event NRI (95% CI)	Referent	0.0786 (-0.0034, 0.1607)	0.0356 (-0.0499, 0.1210)
	Event NRI (95% CI)	Referent	-0.1370 (-0.3220, 0.0480)	-0.1417 (-0.3413, 0.0578)
	Overall NRI (95% CI)	Referent	-0.0584 (-0.3071, 0.1903)	-0.1062 (-0.3725, 0.1601)
Landmark age 80	Non-event NRI (95% CI)	Referent	0.0224 (-0.1638, 0.2086)	0.0877 (-0.0719, 0.2474)
	Event NRI (95% CI)	Referent	-0.0650 (-0.2911, 0.1612)	-0.1982 (-0.3928, -0.0036)
	Overall NRI (95% CI)	Referent	-0.0425 (-0.4351, 0.3500)	-0.1104 (-0.4469, 0.2261)

Abbreviations: CI, confidence interval; NRI, net reclassification improvement.

* NRI was calculated using continuous change in predicted risk. The results are listed in 10-year increments in landmark ages at 40, 50, 60, 70, 80 for presentation.

† Results shown in this table were calculated among type 2 diabetes individuals with **at least one** measurement for each of systolic blood pressure (SBP), total cholesterol, high-density lipoprotein (HDL) cholesterol, and glycated haemoglobin (HbA_{1c}) prior to each landmark age in the validation dataset from 1/3 study population (N=28,285).

‡ Last observed value: Sex-specific Cox regression model that used estimated risk factor values of SBP, total cholesterol, HDL cholesterol, and HbA_{1c} from **last observed values** prior to the landmark age, together with landmark age, landmark age squared, ethnicity, duration of diabetes, smoking status, blood pressure-lowering medication use, and atrial fibrillation status, plus landmark age interaction terms with SBP, total cholesterol, HDL cholesterol, HbA_{1c}, and smoking status.

§ Mean model: Sex-specific Cox regression model that used estimated risk factor values of SBP, total cholesterol, HDL cholesterol, and HbA_{1c} from cumulative mean (**Mean**) of the prior repeated measures, together with other risk factors and interaction terms as same as the last observed value model.

| | Estimated current value model: Sex-specific Cox regression model that used **estimated current risk factor values** at the landmark age of SBP, total cholesterol, HDL cholesterol, and HbA_{1c} from **multivariate mixed-effects linear regression model** of the prior repeated measures, together with other risk factors and interaction terms as same as the last observed value model.

Supplementary Table S10. Changes in 10-year cardiovascular disease risk classification* among people with type 2 diabetes and with at least two measurements in the validation dataset (N = 25,512)†, Clinical Practice Research Datalink, Hospital Episode Statistics, and the Office for National Statistics, England, United Kingdom, 2004-2017

**Men**	**NRI**	**Last observed value model‡**	**Last observed value + SD model§**	**Mean value + SD model||**	**Estimated current value + SD model ¶**
Landmark age 40	Non-event NRI (95% CI)	Referent	0.2794 (0.1229, 0.4359)	0.2623 (0.1252, 0.3995)	0.2310 (0.1042, 0.3577)
	Event NRI (95% CI)	Referent	0.2289 (-0.5143, 0.9721)	0.1198 (-0.5194, 0.7591)	-0.3519 (-0.9312, 0.2275)
	Overall NRI (95% CI)	Referent	0.5083 (-0.3412, 1.3579)	0.3822 (-0.3304, 1.0947)	-0.1209 (-0.7807, 0.5390)
Landmark age 50	Non-event NRI (95% CI)	Referent	0.0340 (-0.0490, 0.1169)	0.0531 (-0.0309, 0.1371)	0.1265 (0.0495, 0.2035)
	Event NRI (95% CI)	Referent	0.1037 (-0.1920, 0.3995)	-0.1031 (-0.3808, 0.1747)	-0.0559 (-0.3544, 0.2425)
	Overall NRI (95% CI)	Referent	0.1377 (-0.2196, 0.4950)	-0.0500 (-0.3855, 0.2856)	0.0706 (-0.2771, 0.4183)
Landmark age 60	Non-event NRI (95% CI)	Referent	0.0218 (-0.0398, 0.0834)	0.0431 (-0.0250, 0.1111)	0.0962 (0.0255, 0.1670)
	Event NRI (95% CI)	Referent	0.0560 (-0.1137, 0.2258)	0.1250 (-0.0480, 0.2979)	0.0121 (-0.1696, 0.1939)
	Overall NRI (95% CI)	Referent	0.0778 (-0.1353, 0.2909)	0.1680 (-0.0552, 0.3912)	0.1084 (-0.1223, 0.3390)
Landmark age 70	Non-event NRI (95% CI)	Referent	-0.0320 (-0.1285, 0.0645)	-0.0471 (-0.1370, 0.0427)	0.0360 (-0.0589, 0.1310)
	Event NRI (95% CI)	Referent	0.1365 (-0.0075, 0.2805)	-0.0642 (-0.1970, 0.0686)	-0.0329 (-0.1844, 0.1187)
	Overall NRI (95% CI)	Referent	0.1044 (-0.1200, 0.3288)	-0.1114 (-0.3194, 0.0966)	0.0032 (-0.2301, 0.2364)
Landmark age 80	Non-event NRI (95% CI)	Referent	0.0518 (-0.1288, 0.2323)	0.1208 (-0.0654, 0.3069)	0.2049 (-0.0020, 0.4118)
	Event NRI (95% CI)	Referent	0.2415 (0.0946, 0.3885)	0.3037 (0.1441, 0.4634)	0.2877 (0.1203, 0.4551)
	Overall NRI (95% CI)	Referent	0.2933 (-0.0127, 0.5992)	0.4245 (0.1004, 0.7486)	0.4926 (0.1396, 0.8456)
**Women**	**NRI**	**Last observed value model**	**Last observed value + SD model**	**Mean + SD model**	**Estimated value + SD model**
Landmark age 40	Non-event NRI (95% CI)	Referent	-0.1541 (-0.4031, 0.0948)	0.0626 (-0.1306, 0.2557)	0.1894 (-0.0340, 0.4127)
	Event NRI (95% CI)	Referent	1.0691 (0.3787, 1.7595)	0.2779 (-0.4103, 0.9661)	0.6227 (-0.0892, 1.3346)
	Overall NRI (95% CI)	Referent	0.9150 (0.0395, 1.7904)	0.3405 (-0.4714, 1.1524)	0.8121 (-0.0443, 1.6684)
Landmark age 50	Non-event NRI (95% CI)	Referent	0.1726 (-0.2760, -0.0691)	0.0627 (-0.0497, 0.1750)	0.0405 (-0.0699, 0.1509)
	Event NRI (95% CI)	Referent	0.3853 (-0.0478, 0.8183)	0.3117 (-0.0377, 0.6611)	0.0793 (-0.3357, 0.4944)
	Overall NRI (95% CI)	Referent	0.2127 (-0.2925, 0.7179)	0.3744 (-0.0504, 0.7992)	0.1198 (-0.3691, 0.6088)
Landmark age 60	Non-event NRI (95% CI)	Referent	0.1892 (-0.2766, -0.1018)	-0.0789 (-0.1608, 0.0030)	-0.0626 (-0.1539, 0.0287)
	Event NRI (95% CI)	Referent	0.0597 (-0.1820, 0.3014)	-0.0311 (-0.2830, 0.2207)	0.0074 (-0.2360, 0.2507)
	Overall NRI (95% CI)	Referent	-0.1295 (-0.4340, 0.1749)	-0.1100 (-0.4186, 0.1986)	-0.0552 (-0.3643, 0.2539)
Landmark age 70	Non-event NRI (95% CI)	Referent	0.0985 (-0.1900, -0.0070)	-0.0345 (-0.1225, 0.0535)	-0.0746 (-0.1690, 0.0198)
	Event NRI (95% CI)	Referent	0.1745 (-0.0089, 0.3580)	0.0160 (-0.1982, 0.2302)	-0.0237 (-0.2277, 0.1802)
	Overall NRI (95% CI)	Referent	0.0760 (-0.1776, 0.3296)	-0.0185 (-0.2987, 0.2616)	-0.0984 (-0.3773, 0.1806)
Landmark age 80	Non-event NRI (95% CI)	Referent	0.2339 (-0.4439, -0.0240)	-0.1314 (-0.3399, 0.0772)	-0.1668 (-0.3831, 0.0495)
	Event NRI (95% CI)	Referent	0.0355 (-0.2214, 0.2923)	0.0826 (-0.1636, 0.3287)	-0.0756 (-0.3311, 0.1799)
	Overall NRI (95% CI)	Referent	-0.1985 (-0.6496, 0.2526)	-0.0488 (-0.4832, 0.3856)	-0.2424 (-0.6942, 0.2094)

Abbreviations: CI, confidence interval; NRI, net reclassification improvement.

* NRI was calculated using continuous change in predicted risk. The results are listed in 10-year increments in landmark ages at 40, 50, 60, 70, 80 for presentation.

† Results shown in this table were calculated among type 2 diabetes individuals with **at least two measurements** for each of systolic blood pressure (SBP), total cholesterol, high-density lipoprotein (HDL) cholesterol, and glycated haemoglobin (HbA_{1c}) prior to each landmark age in the validation dataset from 1/3 study population (N=25,512).

‡ Last observed value model: Sex-specific Cox regression model that used estimated risk factor values of SBP, total cholesterol, HDL cholesterol, and HbA_{1c} from **last observed values** prior to the landmark age, together with landmark age, landmark age squared, ethnicity, duration of diabetes, smoking status, blood pressure-lowering medication use, and atrial fibrillation status, plus landmark age interaction terms with SBP, total cholesterol, HDL cholesterol, HbA_{1c}, and smoking status.

§ Last observed value + SD model: Last observed value model plus individual-level **standard deviations** of SBP, total cholesterol, HDL cholesterol, and HbA_{1c}.

| | Mean value + SD model: Sex-specific Cox regression model that used estimated risk predictor values of SBP, total cholesterol, HDL cholesterol, and HbA_{1c} from cumulative mean (**Mean**) of the historical repeated measures, plus individual-level **standard deviations** of SBP, total cholesterol, HDL cholesterol, and HbA_{1c}, together with other risk predictors and interaction terms as same as the last observed value model.

¶ Estimated current value + SD model: Sex-specific Cox regression model that used **estimated current risk predictor values** of SBP, total cholesterol, HDL cholesterol, and HbA_{1c} from **multivariate mixed-effects linear regression model** of the historical repeated measures, plus individual-level **standard deviations** of SBP, total cholesterol, HDL cholesterol, and HbA_{1c}, together with other risk predictors and interaction terms as same as the last observed value model.

Supplementary Table S11. Hazard ratios* of risk predictors and interaction terms included in the Cox model for cardiovascular disease risk for individuals with type 2 diabetes †, Clinical Practice Research Datalink, Hospital Episode Statistics, and the Office for National Statistics, England, United Kingdom, 2004-2017

**Risk predictor**	**Men**	**Women**
	**HR (95% CI)**	**HR (95% CI)**
Age	1.06 (1.04, 1.07)	1.02 (1.01, 1.04)
Age squared	1.00 (1.00, 1.00)	1.00 (1.00, 1.00)
Duration of diabetes	1.02 (1.02, 1.03)	1.03 (1.03, 1.03)
SBP	1.76 (1.53, 2.02)	1.36 (1.15, 1.61)
Total cholesterol	1.97 (1.76, 2.22)	1.42 (1.24, 1.63)
HDL cholesterol	0.59 (0.53, 0.65)	0.66 (0.58, 0.75)
HbA_{1c}	1.04 (0.92, 1.18)	1.70 (1.50, 1.92)
Current smoking	3.09 (2.39, 4.00)	2.95 (2.13, 4.10)
History of atrial fibrillation	1.81 (1.73, 1.89)	2.29 (2.18, 2.41)
Blood pressure-lowering medication use	1.32 (1.28, 1.36)	1.45 (1.39, 1.51)
Ethnicity		
White	Ref.	Ref.
Mixed	1.30 (1.09, 1.55)	0.80 (0.63, 1.01)
Asian	1.33 (1.26, 1.41)	1.36 (1.28, 1.45)
Black or Black British	0.83 (0.75, 0.91)	0.75 (0.68, 0.84)
Other	1.25 (1.09, 1.42)	1.08 (0.90, 1.28)
Unspecified	0.96 (0.92, 0.99)	1.00 (0.96, 1.04)
Missing	1.04 (1.01, 1.06)	1.06 (1.03, 1.09)
Age×SBP	0.99 (0.99, 1.00)	1.00 (1.00, 1.00)
Age×Total cholesterol	0.99 (0.99, 1.00)	1.00 (1.00, 1.00)
Age×HDL cholesterol	1.00 (1.00, 1.01)	1.00 (1.00, 1.01)
Age×HbA_{1c}	1.00 (1.00, 1.00)	1.00 (0.99, 1.00)
Age×Current smoking	0.99 (0.98, 0.99)	0.99 (0.99, 0.99)

Abbreviations: CI, confidence interval; HDL, high-density lipoprotein; HbA_{1c}, glycated haemoglobin; SBP, systolic blood pressure.

* Hazard ratios are given pre standard deviation increase for SBP, total cholesterol, HDL cholesterol, and HbA_{1c}; and per year increase in duration of type 2 diabetes.

† Models were developed among those with at least 1 measurement of any risk factors of systolic blood pressure, total cholesterol, HDL cholesterol, HbA1C, or smoking status in the derivation dataset (n = 92,585, about 2/3 of the total 143,466). Sex-specific Cox regression models were fitted using **estimated current risk factor values** at the landmark age of SBP, total cholesterol, HDL cholesterol, and HbA_{1c} from **multivariate mixed-effects linear regression models** of the prior repeated measures, together with other risk factors and interaction terms as same as the last observed value model.

Supplementary Table S12. C-index for cardiovascular disease risk prediction for people with type 2 diabetes in the validation dataset*, Clinical Practice Research Datalink, Hospital Episode Statistics, and the Office for National Statistics, England, United Kingdom, 2004-2017

C-index (95% CI)		
Overall	Men	Women
0.670 (0.667, 0.673)	0.665 (0.661, 0.669)	0.675 (0.670, 0.680)

Abbreviations: CI, confidence interval

* Models were developed among those with at least 1 measurement of any risk factors of systolic blood pressure, total cholesterol, HDL cholesterol, HbA1C, or smoking status in the derivation dataset (n = 50,881, about 1/3 of the total 143,466). Sex-specific Cox regression models were fitted using **estimated current risk factor values** at the landmark age of SBP, total cholesterol, HDL cholesterol, and HbA_{1c} from **multivariate mixed-effects linear regression models** of the prior repeated measures, together with other risk factors and interaction terms as same as the last observed value model.

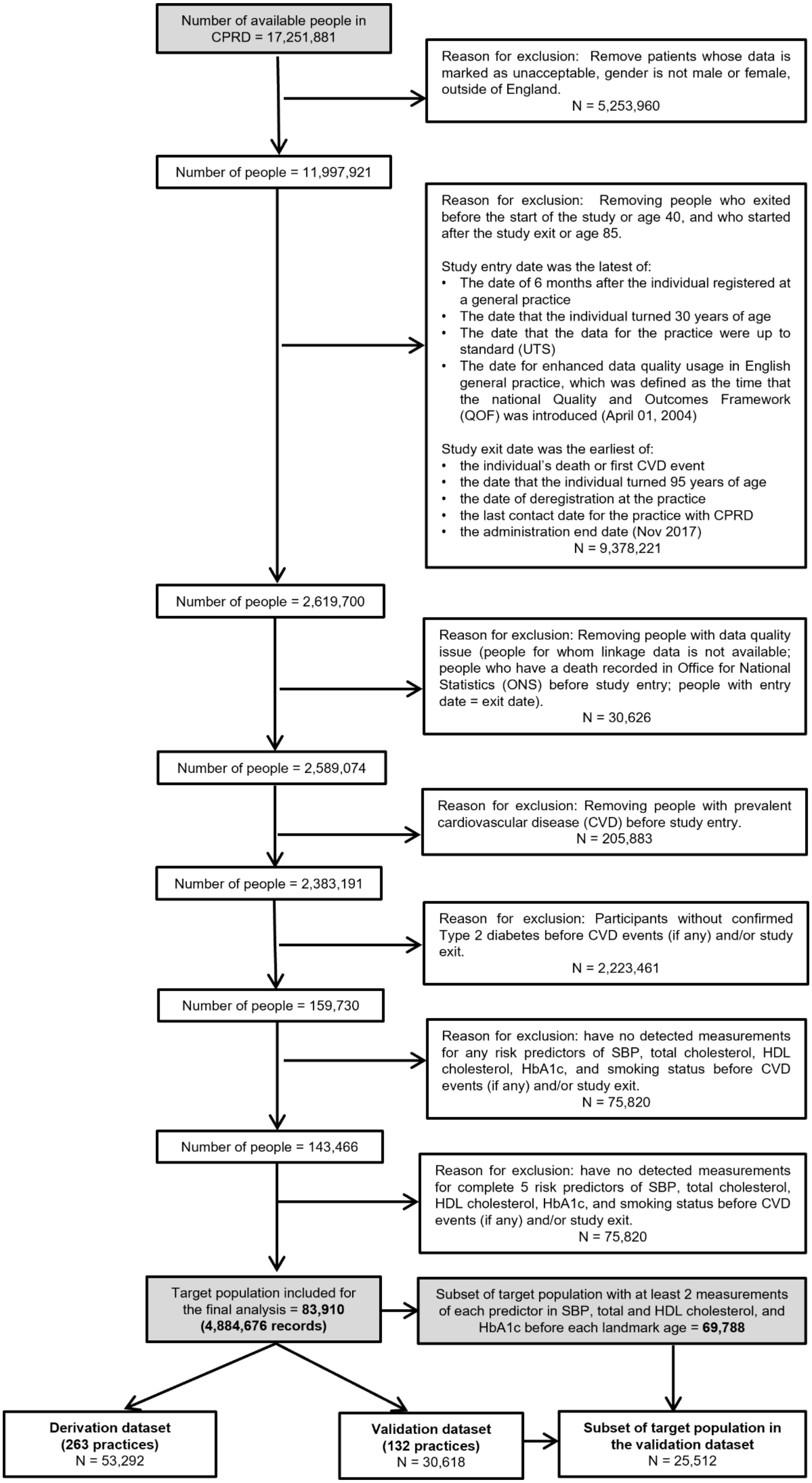

Supplementary Figure S1. Flowchart of individuals included in the study

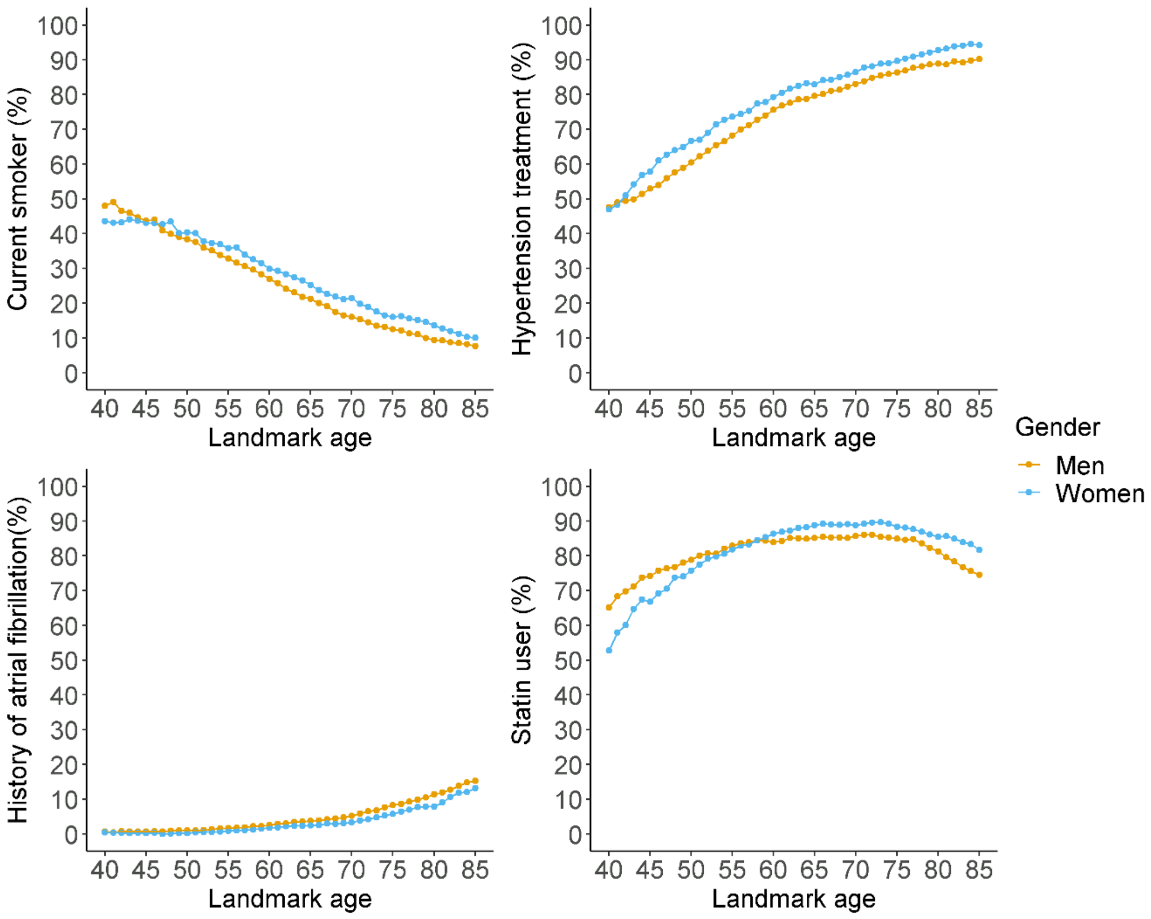

Supplementary Figure S2. The percentage of current smoker, blood pressure-lowering medication use, history of atrial fibrillation, and statin user at each landmark age for people with type 2 diabetes, Clinical Practice Research Datalink, Hospital Episode Statistics, and the Office for National Statistics, England, United Kingdom, 2004-2017

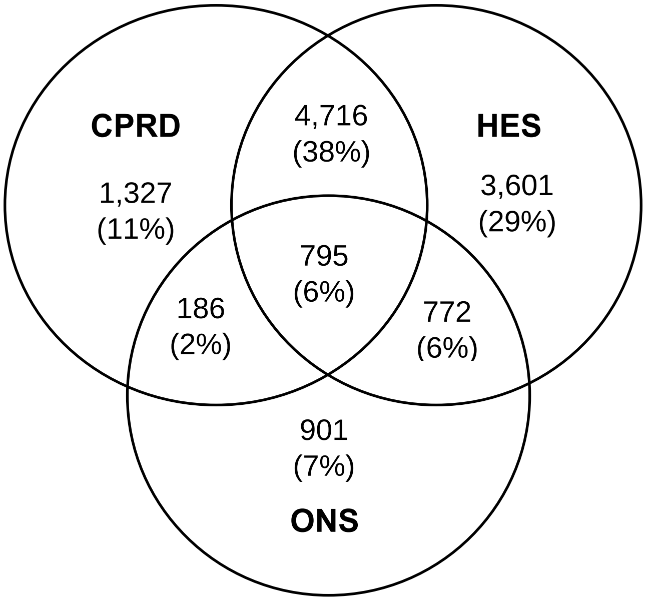

Supplementary Figure S3. Venn diagram of the incident cardiovascular events among people with type 2 diabetes during follow-up recorded from primary care data in Clinical Practice Research Datalink (CPRD) (n=7,024 first events identified), secondary care data in Hospital Episode Statistics (HES) (n=9,884 first events identified), and mortality records in Office for National Statistics (ONS) (n=2,654 first events identified), England, United Kingdom, 2004-2017

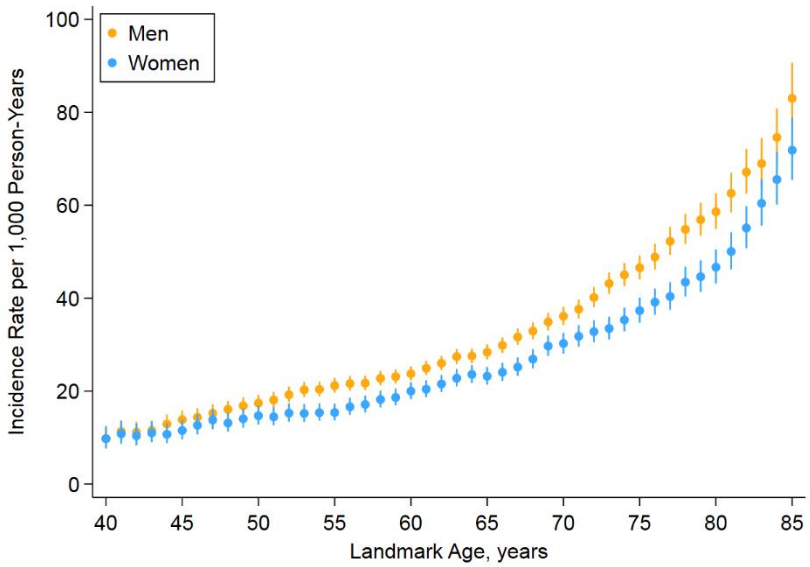

Supplementary Figure S4. Sex-specific 10-year cardiovascular disease incidence rates among people with type 2 diabetes by landmark age, Clinical Practice Research Datalink, Hospital Episode Statistics and the Office for National Statistics, England, United Kingdom, 2004-2017

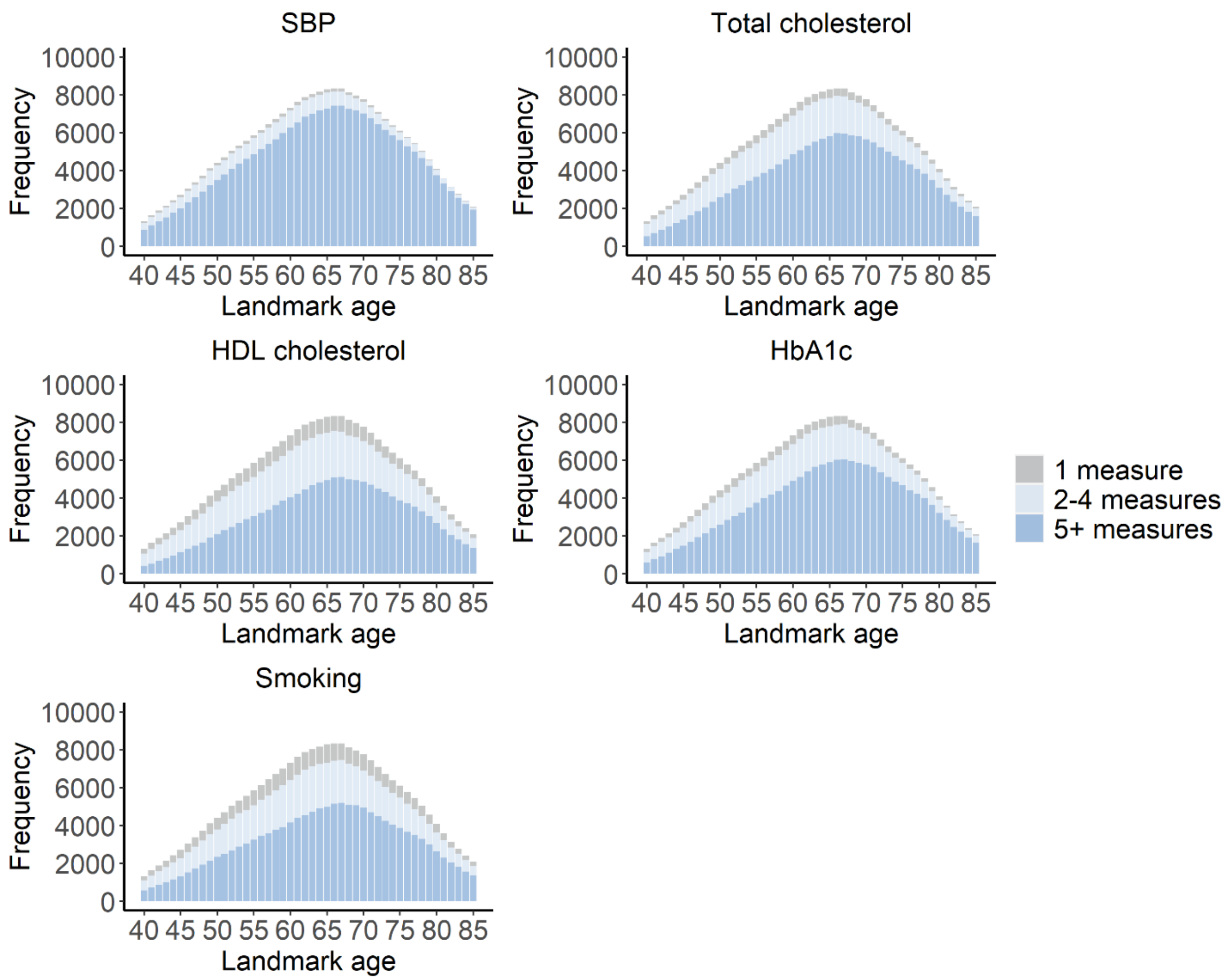

Supplementary Figure S5. Number of measurements of systolic blood pressure (SBP), total cholesterol, high-density lipoprotein (HDL) cholesterol, and glycated haemoglobin (HbA_{1c}) for **men** with type 2 diabetes, Clinical Practice Research Datalink, Hospital Episode Statistics, and the Office for National Statistics, England, United Kingdom, 2004-2017

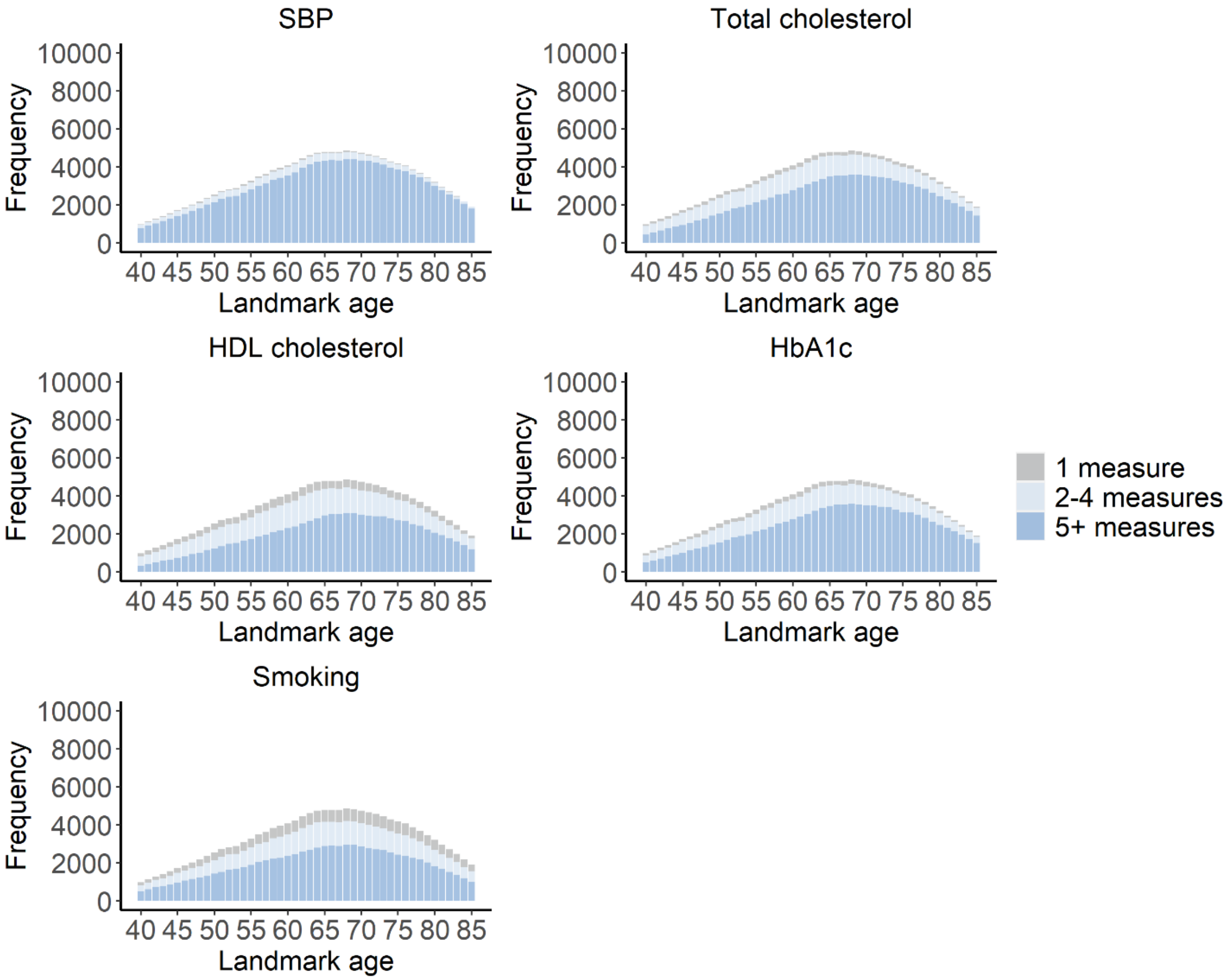

Supplementary Figure S6. Number of measurements of systolic blood pressure (SBP), total cholesterol, high-density lipoprotein (HDL) cholesterol, and glycated haemoglobin (HbA_{1c}) for **women** with type 2 diabetes, Clinical Practice Research Datalink, Hospital Episode Statistics, and the Office for National Statistics, England, United Kingdom, 2004-2017

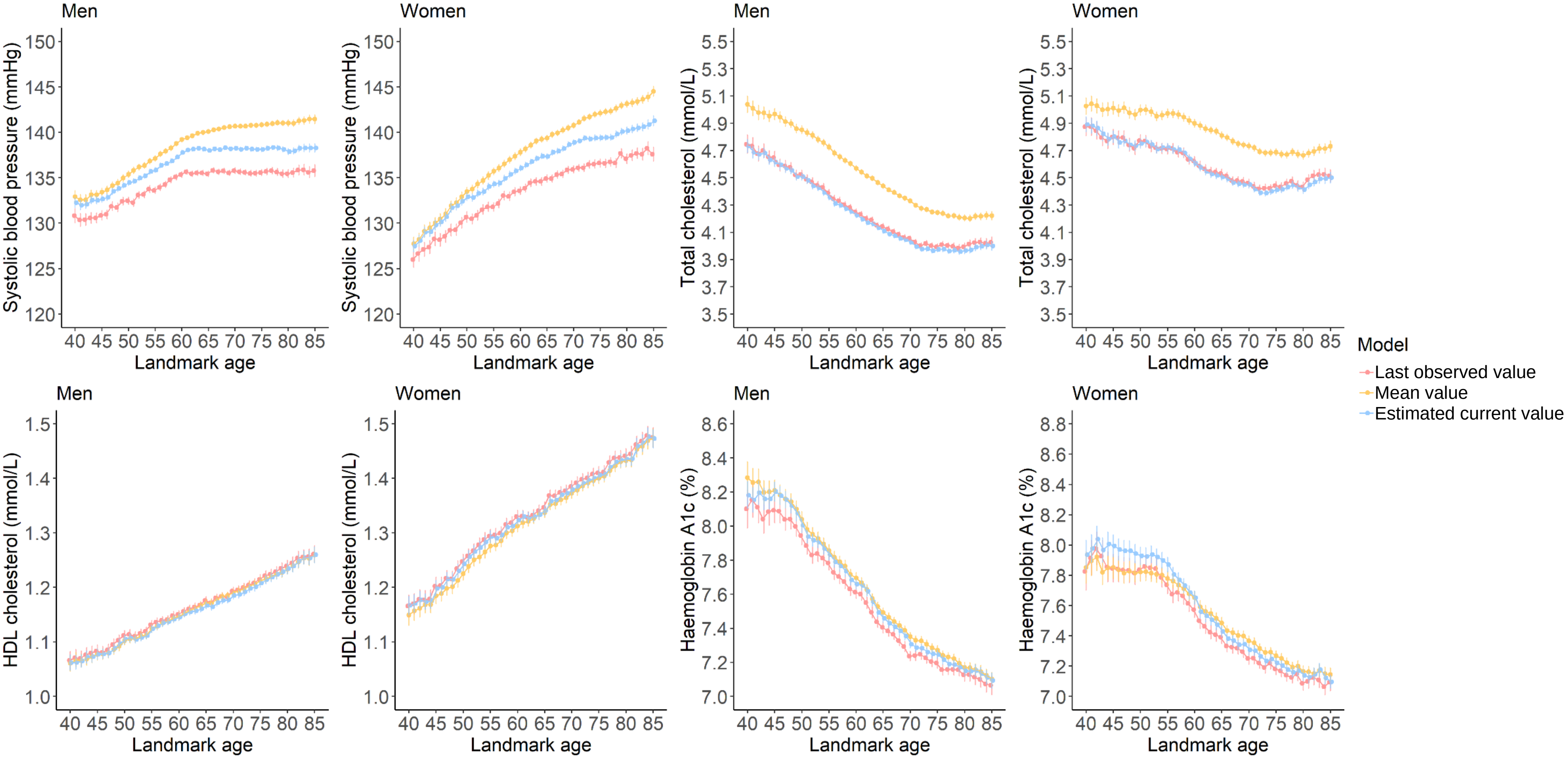

Supplementary Figure S7. The population-level means of risk predictor values of systolic blood pressure (SBP), total cholesterol, high-density lipoprotein (HDL) cholesterol, and glycated haemoglobin (HbA_{1c}) at each landmark age summarised from different models of **last observed values** prior to the landmark age, cumulative **mean values**, and **estimated current values** from multivariate mixed-effects linear regression models for people with type 2 diabetes by landmark age, Clinical Practice Research Datalink, Hospital Episode Statistics and the Office for National Statistics, England, United Kingdom, 2004-2017

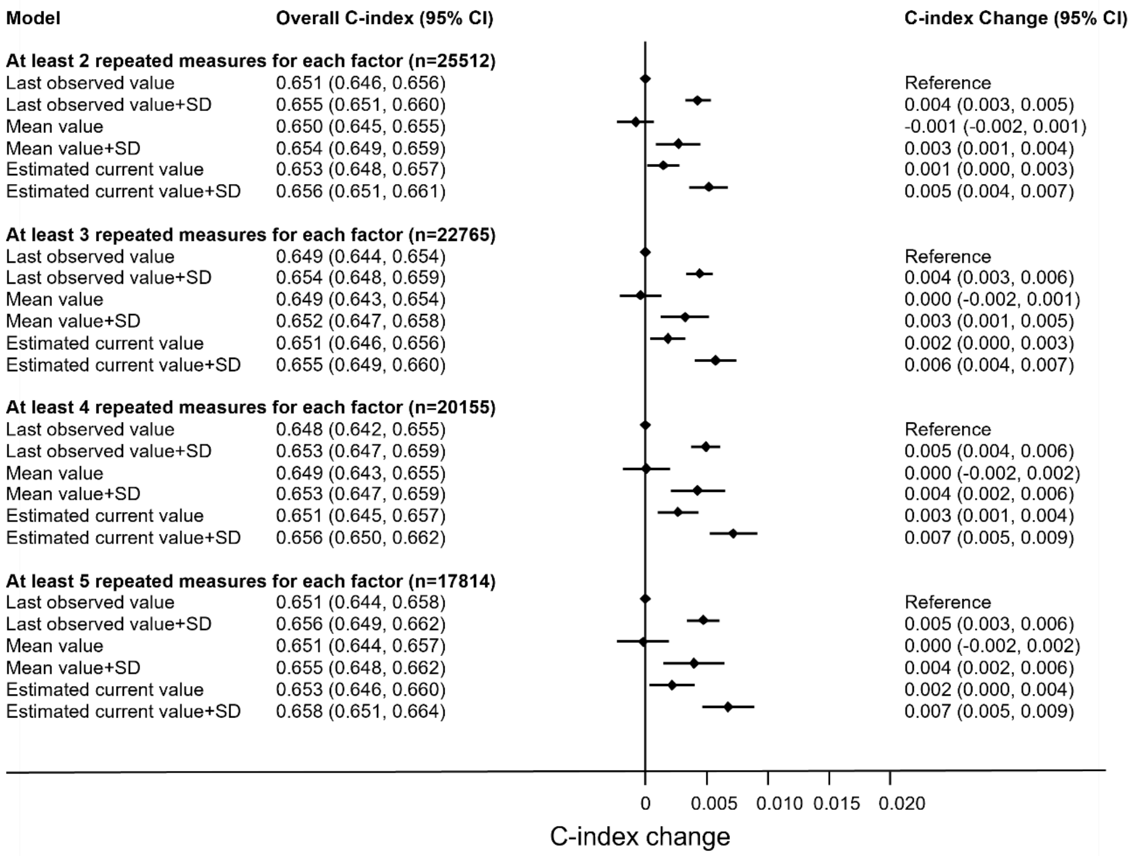

Supplementary Figure S8. Change in cardiovascular disease risk discrimination between models with **standard deviations of systolic blood pressure (SBP)** in the validation for people with type 2 diabetes and with at least two measurements of **each** factor in SBP, total cholesterol, high-density lipoprotein (HDL) cholesterol, and glycated haemoglobin (HbA_{1c}), grouping by the number of repeated measures for each of SBP, total cholesterol, HDL cholesterol, and HbA_{1c}.

Last observed value model: Sex-specific Cox regression model that used estimated risk factor values of SBP, total cholesterol, HDL cholesterol, and HbA_{1c} from **last observed values** prior to the landmark age, together with landmark age, landmark age squared, ethnicity, duration of diabetes, smoking status, blood pressure-lowering medication use, and atrial fibrillation status, plus landmark age interaction terms with SBP, total cholesterol, HDL cholesterol, HbA_{1c}, and smoking status.

Last observed value + SD model: Last observed value model plus individual-level **standard deviations of SBP**.

Mean value model: Sex-specific Cox regression model that used estimated risk factor values of SBP, total cholesterol, HDL cholesterol, and HbA_{1c} from cumulative mean (**Mean**) of the prior repeated measures, together with other risk factors and interaction terms as same as the last observed value model.

Mean value + SD model: Mean value model plus individual-level **standard deviations of SBP**.

Estimated current value model: Sex-specific Cox regression model that used **estimated current risk factor values** at the landmark age of SBP, total cholesterol, HDL cholesterol, and HbA_{1c} from **multivariate mixed-effects linear regression models** of the prior repeated measures, together with other risk factors and interaction terms as same as the last observed value model.

Estimated current value + SD model: Estimated current value model plus individual-level **standard deviations of SBP**.

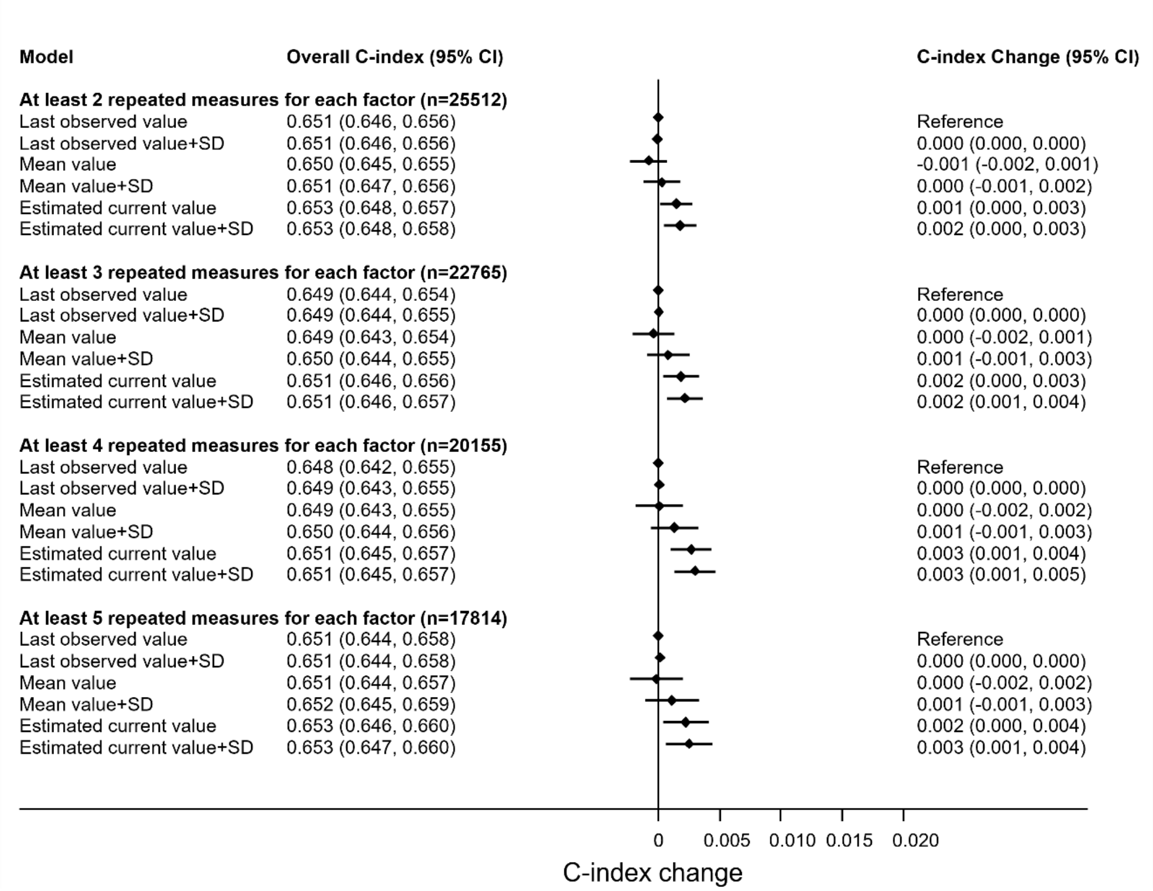

Supplementary Figure S9. Change in cardiovascular disease risk discrimination between models with **standard deviations of total cholesterol** in the validation for people with type 2 diabetes and with at least two measurements of **each** factor in systolic blood pressure (SBP), total cholesterol, high-density lipoprotein (HDL) cholesterol, and glycated haemoglobin (HbA_{1c}), grouping by the number of repeated measures for each of SBP, total cholesterol, HDL cholesterol, and $HbA_{1c.}$

Last observed value model: Sex-specific Cox regression model that used estimated risk factor values of SBP, total cholesterol, HDL cholesterol, and HbA_{1c} from **last observed values** prior to the landmark age, together with landmark age, landmark age squared, ethnicity, duration of diabetes, smoking status, blood pressure-lowering medication use, and atrial fibrillation status, plus landmark age interaction terms with SBP, total cholesterol, HDL cholesterol, HbA_{1c}, and smoking status.

Last observed value + SD model: Last observed value model plus individual-level **standard deviations of total cholesterol.**

Mean value model: Sex-specific Cox regression model that used estimated risk factor values of SBP, total cholesterol, HDL cholesterol, and HbA_{1c} from cumulative mean (**Mean**) of the prior repeated measures, together with other risk factors and interaction terms as same as the last observed value model.

Mean value + SD model: Mean value model plus individual-level **standard deviations of total cholesterol.**

Estimated current value model: Sex-specific Cox regression model that used **estimated current risk factor values** at the landmark age of SBP, total cholesterol, HDL cholesterol, and HbA_{1c} from **multivariate mixed-effects linear regression models** of the prior repeated measures, together with other risk factors and interaction terms as same as the last observed value model.

Estimated current value + SD model: Estimated current value model plus individual-level **standard deviations of total cholesterol.**

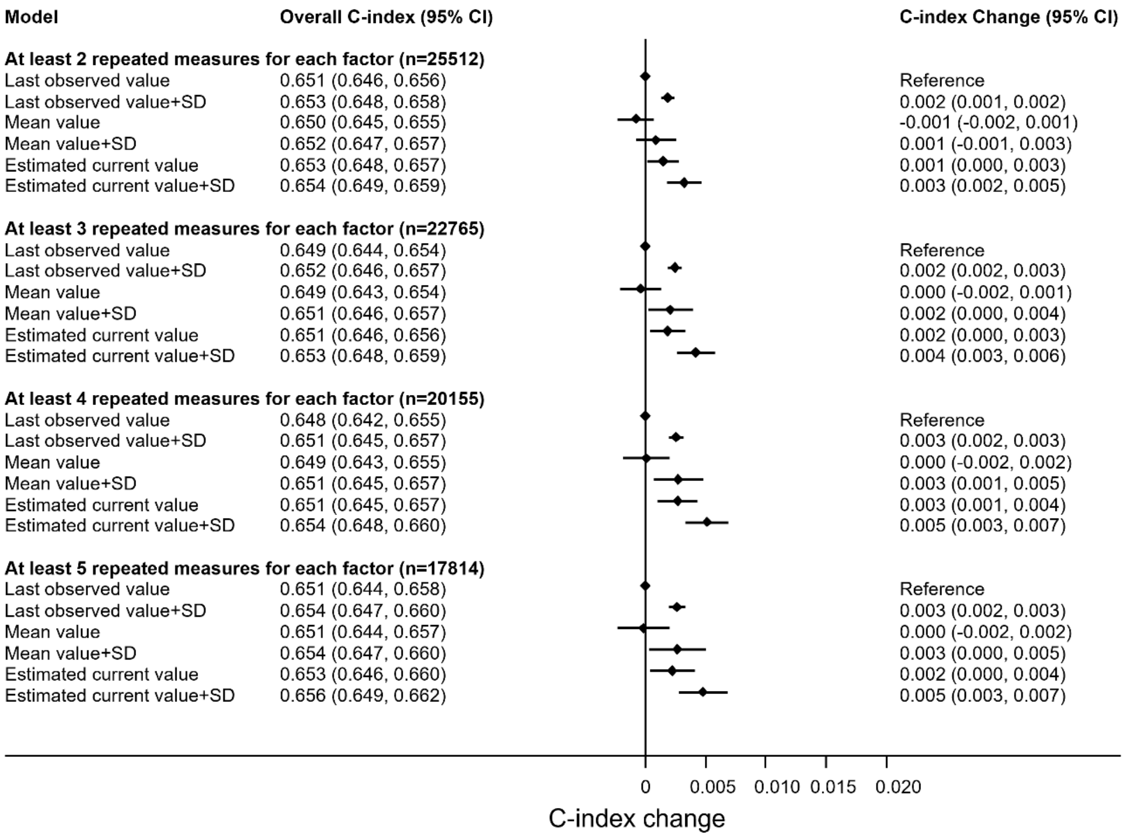

Supplementary Figure S10. Change in cardiovascular disease risk discrimination between models with **standard deviations of high-density lipoprotein (HDL) cholesterol** in the validation for people with type 2 diabetes and with at least two measurements of **each** factor in systolic blood pressure (SBP), total cholesterol, HDL cholesterol, and glycated haemoglobin (HbA_{1c}), grouping by the number of repeated measures for each of SBP, total cholesterol, HDL cholesterol, and HbA_{1c}.

Last observed value model: Sex-specific Cox regression model that used estimated risk factor values of SBP, total cholesterol, HDL cholesterol, and HbA_{1c} from **last observed values** prior to the landmark age, together with landmark age, landmark age squared, ethnicity, duration of diabetes, smoking status, blood pressure-lowering medication use, and atrial fibrillation status, plus landmark age interaction terms with SBP, total cholesterol, HDL cholesterol, HbA_{1c}, and smoking status.

Last observed value + SD model: Last observed value model plus individual-level **standard deviations of HDL cholesterol.**

Mean value model: Sex-specific Cox regression model that used estimated risk factor values of SBP, total cholesterol, HDL cholesterol, and HbA_{1c} from cumulative mean (**Mean**) of the prior repeated measures, together with other risk factors and interaction terms as same as the last observed value model.

Mean value + SD model: Mean value model plus individual-level **standard deviations of HDL cholesterol.**

Estimated current value model: Sex-specific Cox regression model that used **estimated current risk factor values** at the landmark age of SBP, total cholesterol, HDL cholesterol, and HbA_{1c} from **multivariate mixed-effects linear regression models** of the prior repeated measures, together with other risk factors and interaction terms as same as the last observed value model.

Estimated current value + SD model: Estimated current value model plus individual-level **standard deviations of HDL cholesterol.**

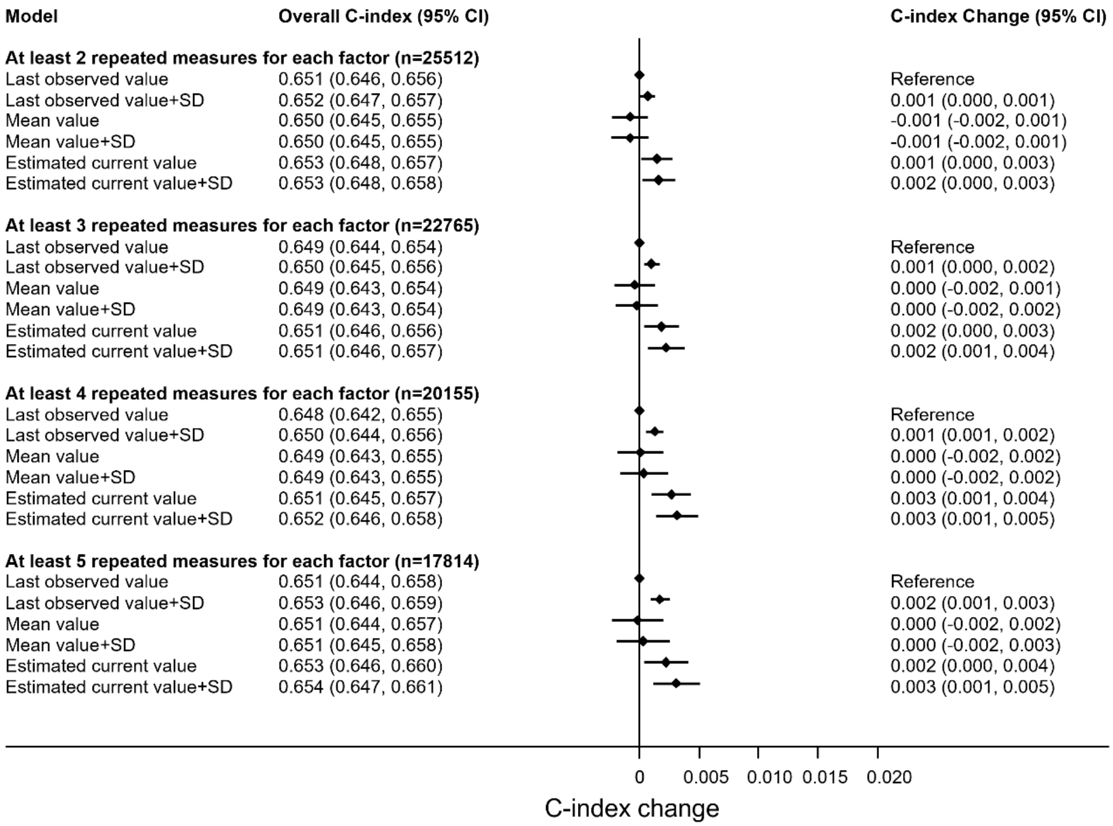

Supplementary Figure S11. Change in cardiovascular disease risk discrimination between models with **standard deviations of glycated haemoglobin (HbA_{1c})** in the validation for people with type 2 diabetes and with at least two measurements of **each** factor in systolic blood pressure (SBP), total cholesterol, high-density lipoprotein (HDL) cholesterol, HbA_{1c}, grouping by the number of repeated measures for each of SBP, total cholesterol, HDL cholesterol, and HbA_{1c}.

Last observed value model: Sex-specific Cox regression model that used estimated risk factor values of SBP, total cholesterol, HDL cholesterol, and HbA_{1c} from **last observed values** prior to the landmark age, together with landmark age, landmark age squared, ethnicity, duration of diabetes, smoking status, blood pressure-lowering medication use, and atrial fibrillation status, plus landmark age interaction terms with SBP, total cholesterol, HDL cholesterol, HbA_{1c}, and smoking status.

Last observed value + SD model: Last observed value model plus individual-level **standard deviations of HbA_{1c}.**

Mean value model: Sex-specific Cox regression model that used estimated risk factor values of SBP, total cholesterol, HDL cholesterol, and HbA_{1c} from cumulative mean (**Mean**) of the prior repeated measures, together with other risk factors and interaction terms as same as the last observed value model.

Mean value + SD model: Mean value model plus individual-level **standard deviations of HbA_{1c}.**

Estimated current value model: Sex-specific Cox regression model that used **estimated current risk factor values** at the landmark age of SBP, total cholesterol, HDL cholesterol, and HbA_{1c} from **multivariate mixed-effects linear regression models** of the prior repeated measures, together with other risk factors and interaction terms as same as the last observed value model.

Estimated current value + SD model: Estimated current value model plus individual-level **standard deviations of HbA_{1c}.**

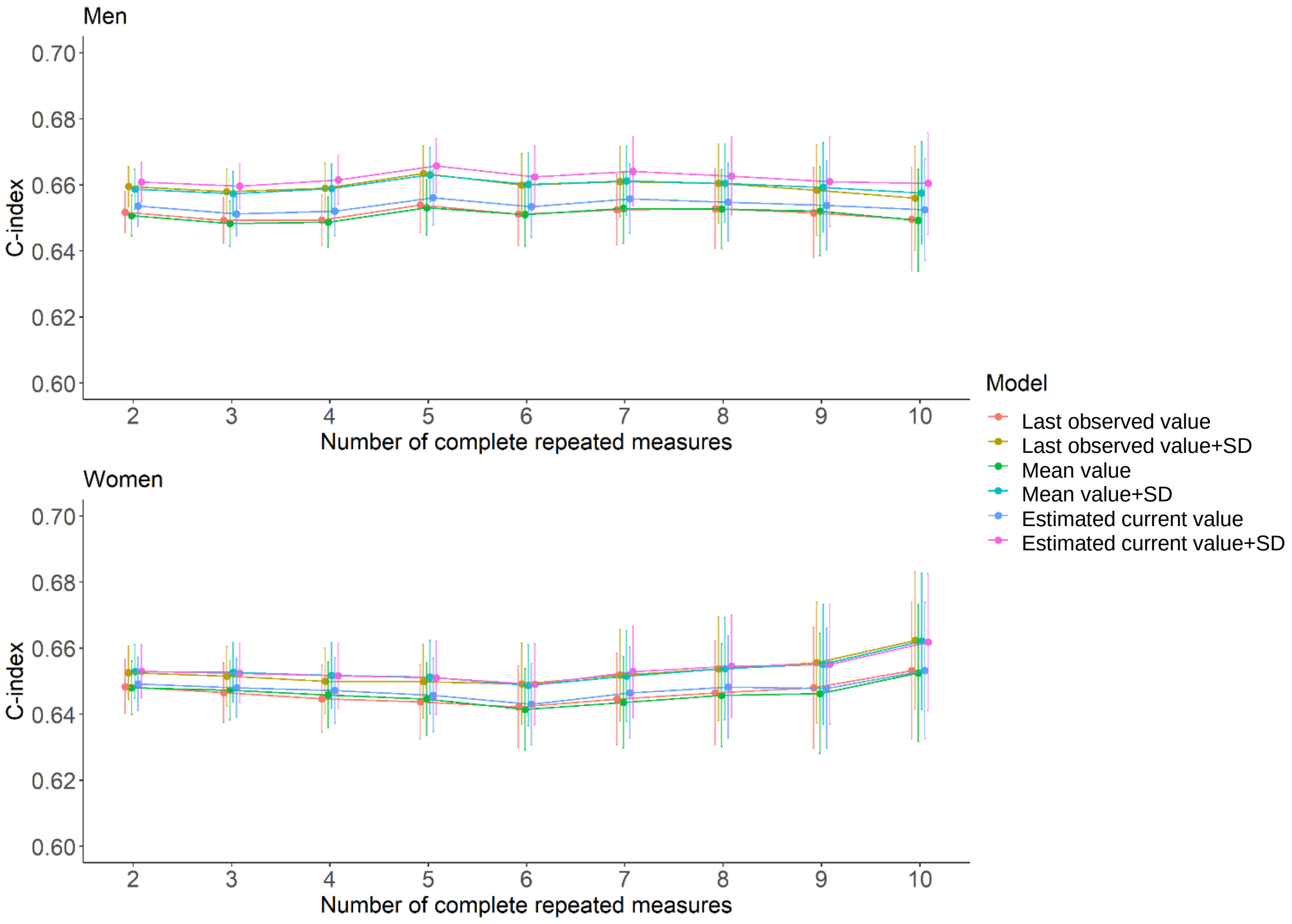

Supplementary Figure S12. Cardiovascular disease risk discrimination between models in the validation for individuals with type 2 diabetes and with **each** factor of systolic blood pressure (SBP), total cholesterol, high-density lipoprotein (HDL) cholesterol, and glycated haemoglobin (HbA_{1c}), grouping by the number of repeated measures for each of SBP, total cholesterol, HDL cholesterol, and HbA_{1c}.

Last observed value model: Sex-specific Cox regression model that used estimated risk factor values of SBP, total cholesterol, HDL cholesterol, and HbA_{1c} from **last observed values** prior to the landmark age, together with landmark age, landmark age squared, ethnicity, duration of diabetes, smoking status, blood pressure-lowering medication use, and atrial fibrillation status, plus landmark age interaction terms with SBP, total cholesterol, HDL cholesterol, HbA_{1c}, and smoking status.

Last observed value + SD model: Last observed value model plus individual-level **standard deviations** of SBP, total cholesterol, HDL cholesterol, and HbA_{1c}.

Mean value model: Sex-specific Cox regression model that used estimated risk factor values of SBP, total cholesterol, HDL cholesterol, and HbA_{1c} from cumulative mean (**Mean**) of the prior repeated measures, together with other risk factors and interaction terms as same as the last observed value model.

Mean value + SD model: Mean value model plus individual-level **standard deviations** of SBP, total cholesterol, HDL cholesterol, and HbA_{1c}.

Estimated current value model: Sex-specific Cox regression model that used **estimated current risk factor values** at the landmark age of SBP, total cholesterol, HDL cholesterol, and HbA_{1c} from **multivariate mixed-effects linear regression models** of the prior repeated measures, together with other risk factors and interaction terms as same as the last observed value model.

Estimated current value + SD model: Estimated current value model plus individual-level **standard deviations** of SBP, total cholesterol, HDL cholesterol, and HbA_{1c}.

Model	Overall C-index (95% CI)	C-index Change (95% CI)
**At least 2 repeated measures for each factor (n=15424)**		
Last observed value	0.652 (0.646, 0.658)	Reference
Last observed value+SD	0.660 (0.653, 0.666)	0.008 (0.006, 0.010)
Mean value	0.651 (0.645, 0.657)	-0.001 (-0.003, 0.001)
Mean value+SD	0.659 (0.653, 0.665)	0.007 (0.004, 0.009)
Estimated current value	0.654 (0.648, 0.660)	0.002 (0.000, 0.004)
Estimated current value+SD	0.661 (0.655, 0.667)	0.009 (0.007, 0.011)
**At least 3 repeated measures for each factor (n=13800)**		
Last observed value	0.649 (0.643, 0.656)	Reference
Last observed value+SD	0.658 (0.651, 0.665)	0.009 (0.007, 0.011)
Mean value	0.648 (0.642, 0.655)	-0.001 (-0.003, 0.001)
Mean value+SD	0.657 (0.651, 0.664)	0.008 (0.005, 0.011)
Estimated current value	0.651 (0.645, 0.658)	0.002 (0.000, 0.004)
Estimated current value+SD	0.660 (0.653, 0.666)	0.010 (0.008, 0.013)
**At least 4 repeated measures for each factor (n=12244)**		
Last observed value	0.649 (0.642, 0.657)	Reference
Last observed value+SD	0.659 (0.652, 0.667)	0.010 (0.008, 0.012)
Mean value	0.649 (0.641, 0.656)	-0.001 (-0.003, 0.002)
Mean value+SD	0.659 (0.651, 0.666)	0.010 (0.006, 0.013)
Estimated current value	0.652 (0.645, 0.660)	0.003 (0.001, 0.005)
Estimated current value+SD	0.662 (0.654, 0.669)	0.012 (0.009, 0.015)
**At least 5 repeated measures for each factor (n=10831)**		
Last observed value	0.654 (0.646, 0.662)	Reference
Last observed value+SD	0.663 (0.655, 0.672)	0.010 (0.007, 0.012)
Mean value	0.653 (0.645, 0.662)	-0.001 (-0.004, 0.002)
Mean value+SD	0.663 (0.655, 0.671)	0.009 (0.006, 0.013)
Estimated current value	0.656 (0.648, 0.664)	0.002 (0.000, 0.005)
Estimated current value+SD	0.666 (0.658, 0.674)	0.012 (0.009, 0.015)

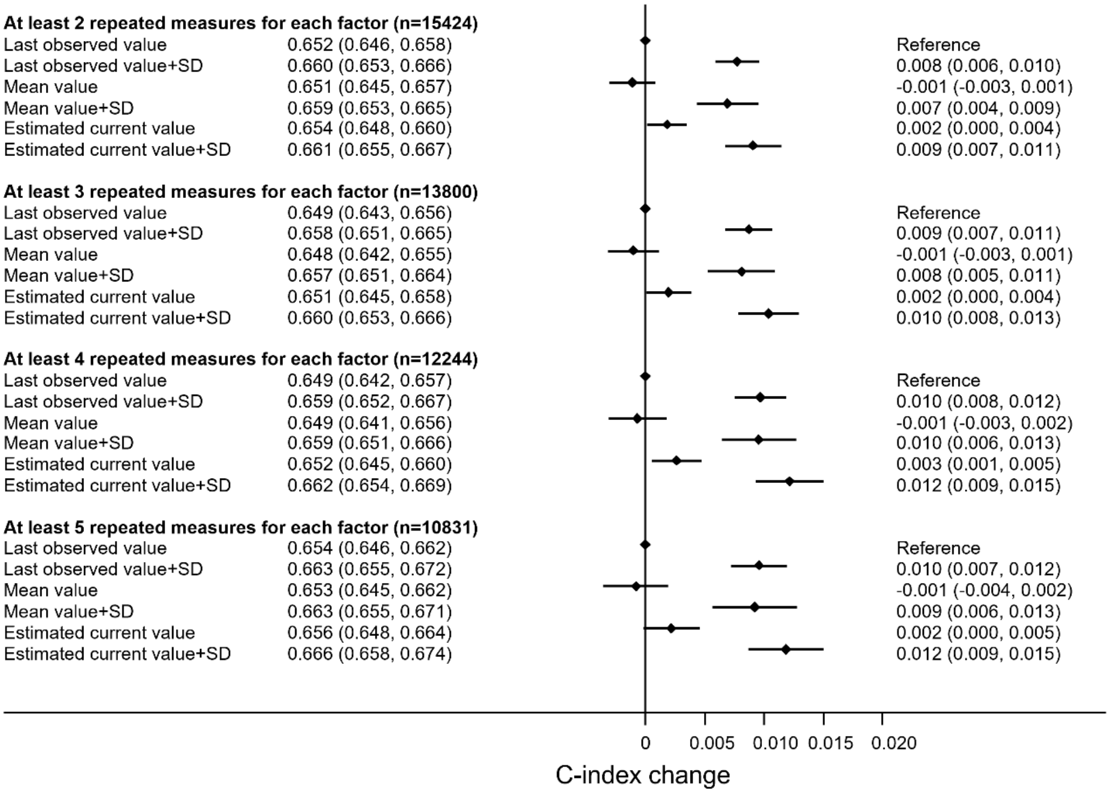

Supplementary Figure S13. Change in cardiovascular disease risk discrimination between models in the validation for **men** with type 2 diabetes and with at least two measurements of **each** factor of systolic blood pressure (SBP), total cholesterol, high-density lipoprotein (HDL) cholesterol, and glycated haemoglobin (HbA_{1c}), grouping by the number of repeated measures for each of SBP, total cholesterol, HDL cholesterol, and HbA_{1c}.

Last observed value model: Sex-specific Cox regression model that used estimated risk factor values of SBP, total cholesterol, HDL cholesterol, and HbA_{1c} from **last observed values** prior to the landmark age, together with landmark age, landmark age squared, ethnicity, duration of diabetes, smoking status, blood pressure-lowering medication use, and atrial fibrillation status, plus landmark age interaction terms with SBP, total cholesterol, HDL cholesterol, HbA_{1c}, and smoking status.

Last observed value + SD model: Last observed value model plus individual-level **standard deviations** of SBP, total cholesterol, HDL cholesterol, and HbA_{1c}.

Mean value model: Sex-specific Cox regression model that used estimated risk factor values of SBP, total cholesterol, HDL cholesterol, and HbA_{1c} from cumulative mean (**Mean**) of the prior repeated measures, together with other risk factors and interaction terms as same as the last observed value model.

Mean value + SD model: Mean value model plus individual-level **standard deviations** of SBP, total cholesterol, HDL cholesterol, and HbA_{1c}.

Estimated current value model: Sex-specific Cox regression model that used **estimated current risk factor values** at the landmark age of SBP, total cholesterol, HDL cholesterol, and HbA_{1c} from **multivariate mixed-effects linear regression models** of the prior repeated measures, together with other risk factors and interaction terms as same as the last observed value model.

Estimated current value + SD model: Estimated current value model plus individual-level **standard deviations** of SBP, total cholesterol, HDL cholesterol, and HbA_{1c}.

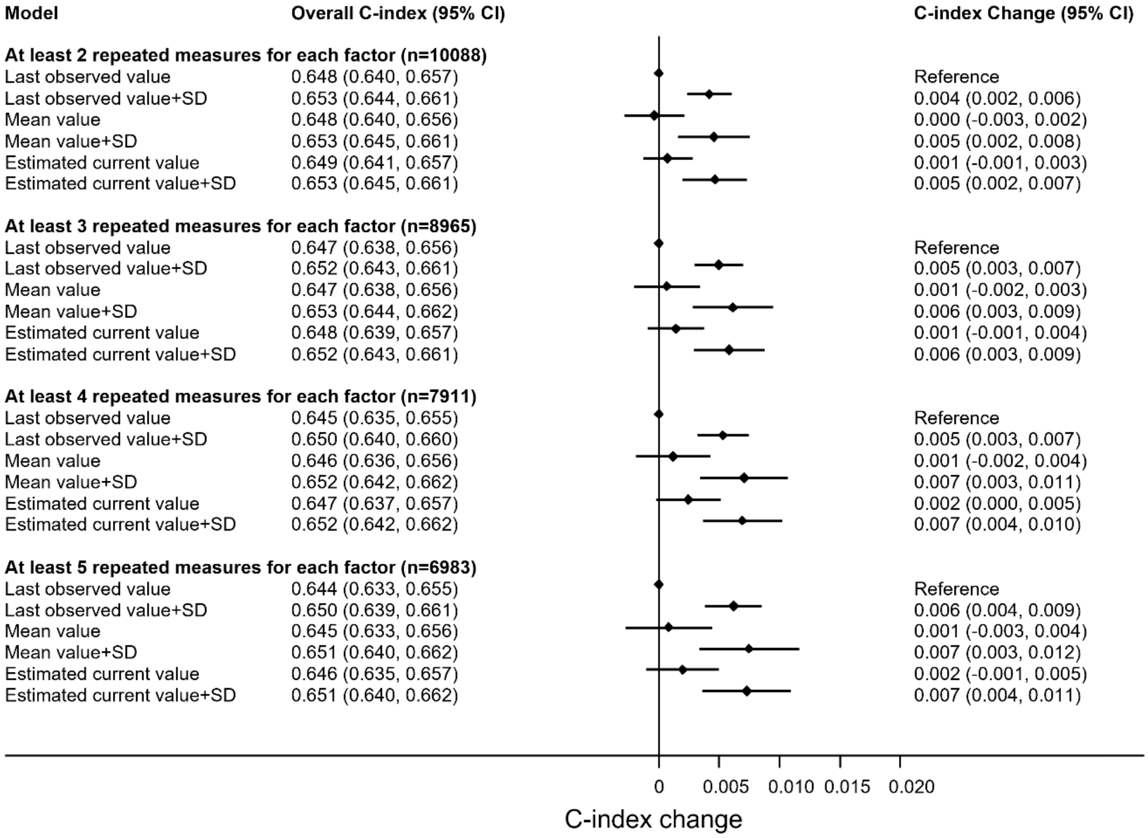

Supplementary Figure S14. Change in cardiovascular disease risk discrimination between models in the validation for **women** with type 2 diabetes and with at least two measurements of **each** factor of systolic blood pressure (SBP), total cholesterol, high-density lipoprotein (HDL) cholesterol, and glycated haemoglobin (HbA_{1c}), grouping by the number of repeated measures for each of SBP, total cholesterol, HDL cholesterol, and HbA_{1c}.

Last observed value model: Sex-specific Cox regression model that used estimated risk factor values of SBP, total cholesterol, HDL cholesterol, and HbA_{1c} from **last observed values** prior to the landmark age, together with landmark age, landmark age squared, ethnicity, duration of diabetes, smoking status, blood pressure-lowering medication use, and atrial fibrillation status, plus landmark age interaction terms with SBP, total cholesterol, HDL cholesterol, HbA_{1c}, and smoking status.

Last observed value + SD model: Last observed value model plus individual-level **standard deviations** of SBP, total cholesterol, HDL cholesterol, and HbA_{1c}.

Mean value model: Sex-specific Cox regression model that used estimated risk factor values of SBP, total cholesterol, HDL cholesterol, and HbA_{1c} from cumulative mean (**Mean**) of the prior repeated measures, together with other risk factors and interaction terms as same as the last observed value model.

Mean value + SD model: Mean value model plus individual-level **standard deviations** of SBP, total cholesterol, HDL cholesterol, and HbA_{1c}.

Estimated current value model: Sex-specific Cox regression model that used **estimated current risk factor values** at the landmark age of SBP, total cholesterol, HDL cholesterol, and HbA_{1c} from **multivariate mixed-effects linear regression models** of the prior repeated measures, together with other risk factors and interaction terms as same as the last observed value model.

Estimated current value + SD model: Estimated current value model plus individual-level **standard deviations** of SBP, total cholesterol, HDL cholesterol, and HbA_{1c}.

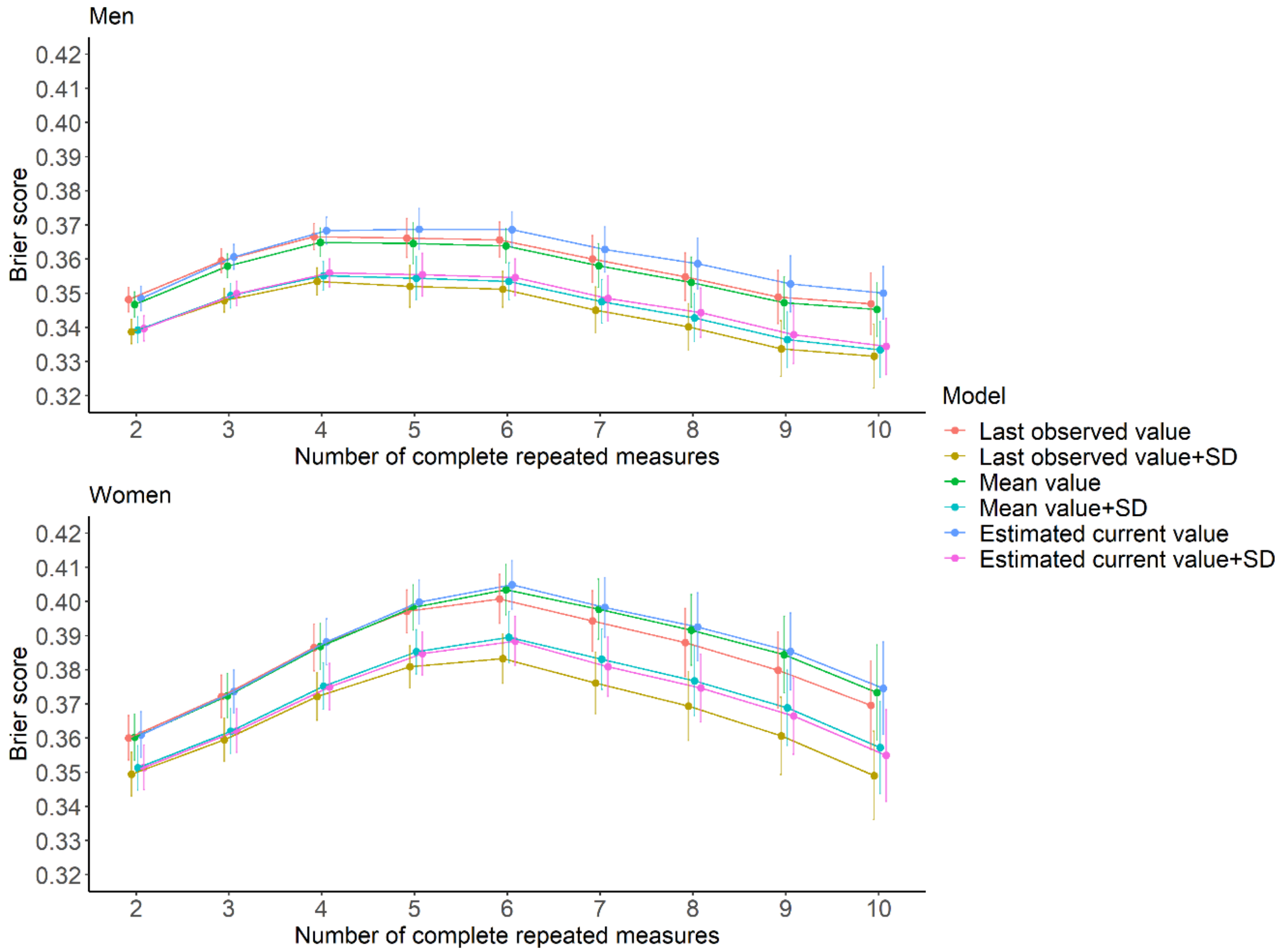

Supplementary Figure S15. Brier scores of the models estimated in the validation for individuals with type 2 diabetes and with **each** factor of systolic blood pressure (SBP), total cholesterol, high-density lipoprotein (HDL) cholesterol, and glycated haemoglobin (HbA_{1c}), grouping by the number of repeated measures for each of SBP, total cholesterol, HDL cholesterol, and HbA_{1c}.

Last observed value model: Sex-specific Cox regression model that used estimated risk factor values of SBP, total cholesterol, HDL cholesterol, and HbA_{1c} from **last observed values** prior to the landmark age, together with landmark age, landmark age squared, ethnicity, duration of diabetes, smoking status, blood pressure-lowering medication use, and atrial fibrillation status, plus landmark age interaction terms with SBP, total cholesterol, HDL cholesterol, HbA_{1c}, and smoking status.

Last observed value + SD model: Last observed value model plus individual-level **standard deviations** of SBP, total cholesterol, HDL cholesterol, and HbA_{1c}.

Mean value model: Sex-specific Cox regression model that used estimated risk factor values of SBP, total cholesterol, HDL cholesterol, and HbA_{1c} from cumulative mean (**Mean**) of the prior repeated measures, together with other risk factors and interaction terms as same as the last observed value model.

Mean value + SD model: Mean value model plus individual-level **standard deviations** of SBP, total cholesterol, HDL cholesterol, and HbA_{1c}.

Estimated current value model: Sex-specific Cox regression model that used **estimated current risk factor values** at the landmark age of SBP, total cholesterol, HDL cholesterol, and HbA_{1c} from **multivariate mixed-effects linear regression models** of the prior repeated measures, together with other risk factors and interaction terms as same as the last observed value model.

Estimated current value + SD model: Estimated current value model plus individual-level **standard deviations** of SBP, total cholesterol, HDL cholesterol, and HbA_{1c}.

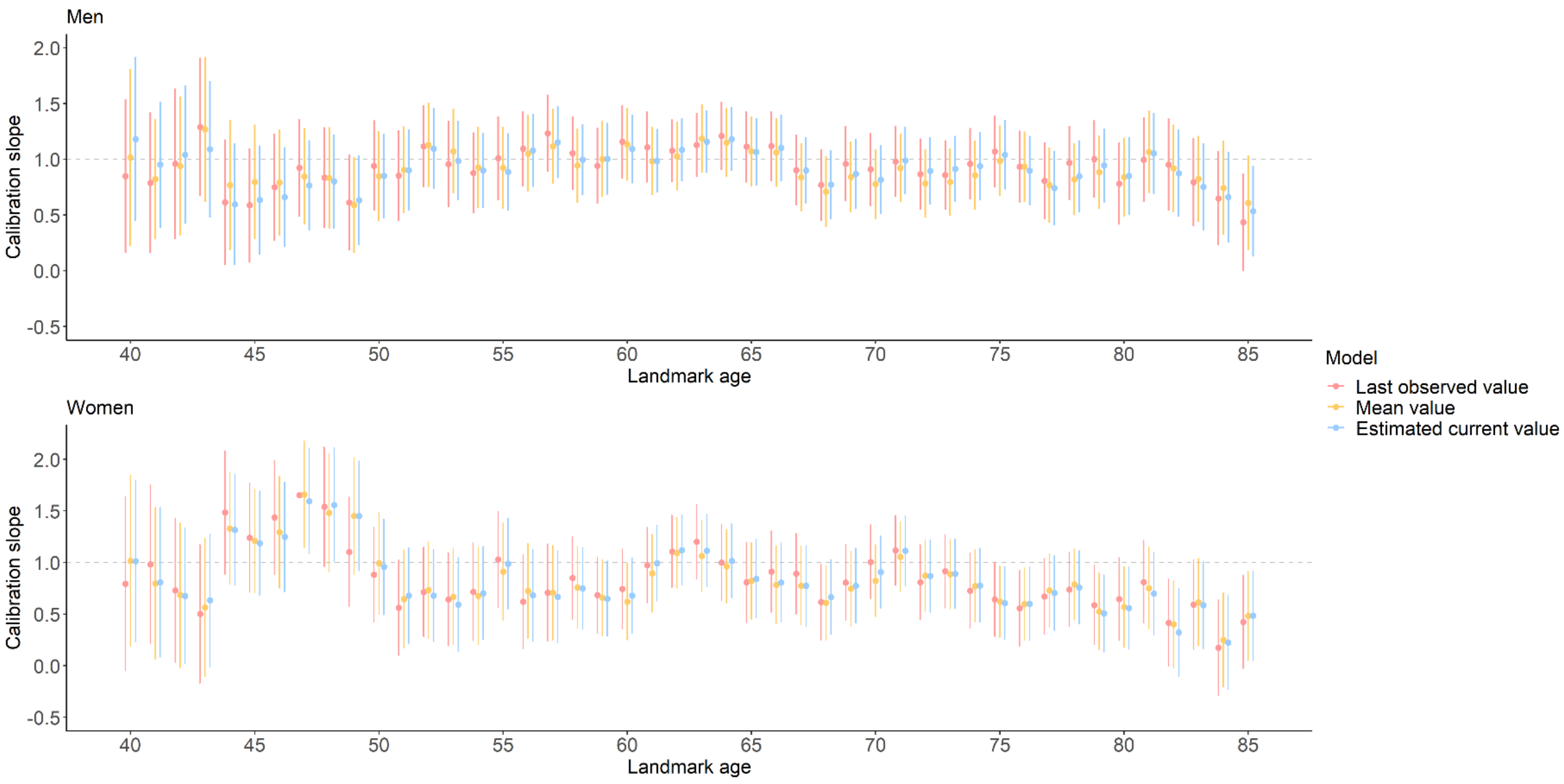

Supplementary Figure S16. Calibration slopes for men and women with type 2 diabetes by landmark age in the validation dataset. Results shown in this figure were calculated among type 2 diabetes individuals with **at least one** measurement for each of systolic blood pressure (SBP), total cholesterol, high-density lipoprotein (HDL) cholesterol, and glycated haemoglobin (HbA_{1c}) prior to each landmark age in the validation dataset from 1/3 study population **(N=28,285)**. Risk was predicted from sex-specific Cox regression models that used estimated risk predictor values of SBP, total cholesterol, HDL cholesterol, and HbA_{1c} from **last observed values** prior to the landmark age, cumulative **mean values**, and **estimated current values** from multivariate mixed-effects linear regression models, together with landmark age, landmark age squared, ethnicity, duration of diabetes, smoking status, blood pressure-lowering medication use, and atrial fibrillation status, plus landmark age interaction terms with SBP, total cholesterol, HDL cholesterol, HbA_{1c}, and smoking status.

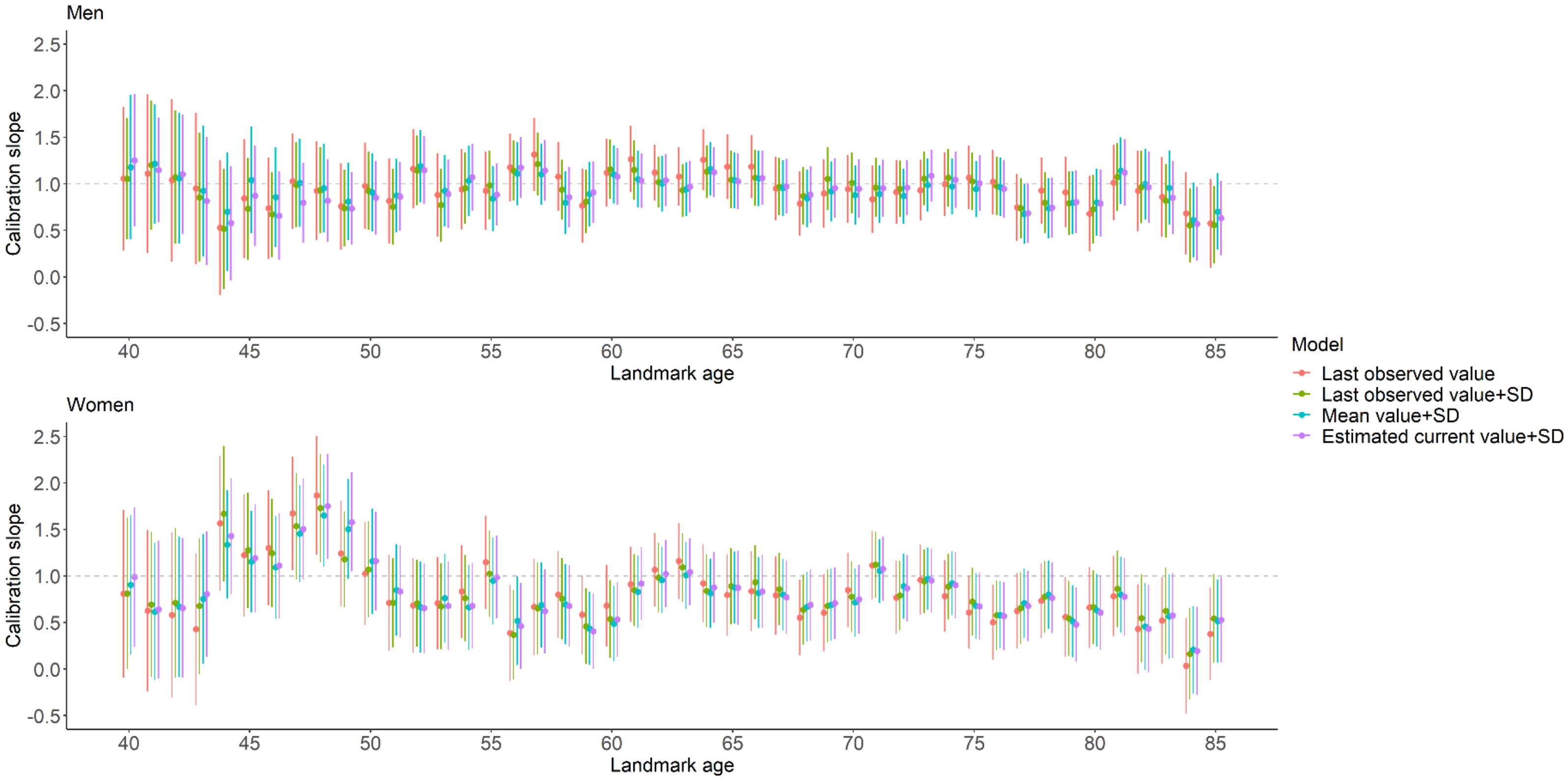

Supplementary Figure S17. Calibration slopes for men and women with type 2 by landmark age in the validation dataset. Results shown were calculated among type 2 diabetes individuals with **at least two measurements** for each of systolic blood pressure (SBP), total cholesterol, high-density lipoprotein (HDL) cholesterol, and glycated haemoglobin (HbA_{1c}) prior to each landmark age in the validation dataset from 1/3 study population **(N=25,512)**. Risk was predicted from sex-specific Cox regression models that used estimated risk predictor values of SBP, total cholesterol, HDL cholesterol, and HbA_{1c} from **last observed values** prior to the landmark age, cumulative **mean values**, and **estimated current values** from multivariate mixed-effects linear regression models, plus individual-level **standard deviations** of SBP, total cholesterol, HDL cholesterol, and HbA_{1c}, together with landmark age, landmark age squared, ethnicity, duration of diabetes, smoking status, blood pressure-lowering medication use, and atrial fibrillation status, plus landmark age interaction terms with SBP, total cholesterol, HDL cholesterol, HbA_{1c}, and smoking status.

References

1. Sharma M, Petersen I, Nazareth I, Coton SJ. An algorithm for identification and classification of individuals with type 1 and type 2 diabetes mellitus in a large primary care database. *Clin Epidemiol* 2016;**8**:373–380.

2. Smolina K, Wotton CJ, Goldacre MJ. Risk of dementia in patients hospitalised with type 1 and type 2 diabetes in England, 1998–2011: a retrospective national record linkage cohort study. *Diabetologia* 2015;**58**:942–950.

3. Hippisley-Cox J, Coupland C, Brindle P. Development and validation of QRISK3 risk prediction algorithms to estimate future risk of cardiovascular disease: prospective cohort study. *BMJ* 2017;**357**:j2099.

4. Herrett E, Shah AD, Boggon R et al. Completeness and diagnostic validity of recording acute myocardial infarction events in primary care, hospital care, disease registry, and national mortality records: cohort study. *BMJ* 2013;**346**:f2350.

5. Herrett E, Gallagher AM, Bhaskaran K et al. Data resource profile: Clinical Practice Research Datalink (CPRD). *Int J Epidemiol* 2015;**44**(3):827–836.

6. Paige E, Barrett J, Stevens D et al. Landmark models for optimizing the use of repeated measurements of risk factors in electronic health records to predict future disease risk. *Am J Epidemiol* 2018;**187**:1530–1538.

7. Verbeke G, Fieuws S, Molenberghs G, Davidian M. The analysis of multivariate longitudinal data: A review. *Stat Methods Med Res* 2014;**23**:42–59.

8. Hajifathalian K, Ueda P, Lu Y et al. A novel risk score to predict cardiovascular disease risk in national populations (Globorisk): a pooled analysis of prospective cohorts and health examination surveys. *Lancet Diabetes Endocrinol* 2015;**3**:339–355.

9. Littman AJ, Boyko EJ, McDonell MB, Fihn SD. Evaluation of a weight management program for veterans. *Prev Chronic Dis* 2012;**9**:E99.

10. Nicholas J, Charlton J, Dregan A, Gulliford MC. Recent HbA1c values and mortality risk in type 2 diabetes. Population-Based Case-Control Study. *PLOS ONE* 2013;**8**:e68008.

11. Goldberger AS. Best linear unbiased prediction in the generalized linear regression model. *Journal of the American Statistical Association* 1962;**57**:369–375.

12. Goldstein H. Multilevel statistical models, 4th edition. New Jersey: Wiley, 2010.

13. Diggle P, Heagerty P, Liang K-Y, Zeger S. Analysis of longitudinal data, second edition. Oxford: Oxford University Press, 2002.

14. van Houwelingen H, Putter H. Dynamic prediction in clinical survival analysis. Boca Raton: CRC Press, 2011.

15. Berkelmans GFN, Gudbjörnsdottir S, Visseren FLJ, et al. Prediction of individual life-years gained without cardiovascular events from lipid, blood pressure, glucose, and aspirin treatment based on data of more than 500 000 patients with Type 2 diabetes mellitus. *Eur Heart J* 2019;**40**:2899-2906.

16. Austin PC, Steyerberg EW. Events per variable (EPV) and the relative performance of different strategies for estimating the out-of-sample validity of logistic regression models. *Stat Methods Med Res* 2017;**26**:796–808.

17. Steyerberg E. Clinical prediction models: a practical approach to development, validation, and updating. New York: Springer, 2009.

18. Harrell FE, Lee KL, Mark DB. Multivariable prognostic models: issues in developing models, evaluating assumptions and adequacy, and measuring and reducing errors. *Statistics in Medicine* 1996;**15**:361–387.

19. Steyerberg EW, Vickers AJ, Cook NR, et al. Assessing the performance of prediction models: a framework for traditional and novel measures. *Epidemiology* 2010;**21**:128–138.

20. Demler OV, Paynter NP, Cook NR. Tests of calibration and goodness of fit in the survival setting. *Stat Med* 2015;**34**:1659–1680.

21. Pencina MJ, D'Agostino RB, Steyerberg EW. Extensions of net reclassification improvement calculations to measure usefulness of new biomarkers. *Stat Med* 2011;**30**:11–21.

22. Collins GS, Reitsma JB, Altman DG, Moons KGM. Transparent Reporting of a multivariable prediction model for Individual Prognosis Or Diagnosis (TRIPOD): The TRIPOD statement. *Ann Intern Med* 2015;**162**:55–63.
